# VASH2 enhances KIF3C-mediated EGFR-endosomal recycling to promote aggression and chemoresistance of lung squamous cell carcinoma by increasing tubulin detyrosination

**DOI:** 10.1038/s41419-024-07155-x

**Published:** 2024-10-23

**Authors:** Jing Wang, Pengpeng Liu, Rui Zhang, Biyuan Xing, Guidong Chen, Lei Han, Jinpu Yu

**Affiliations:** 1 Cancer Molecular Diagnostics Core, Tianjin Medical University Cancer Institute and Hospital, National Clinical Research Center for Cancer, Tianjin’s Clinical Research Center for Cancer, 300202 Tianjin, China; 2 Key Laboratory of Cancer Immunology and Biotherapy, Tianjin Medical University Cancer Institute and Hospital, National Clinical Research Center for Cancer, Tianjin’s Clinical Research Center for Cancer, 300202 Tianjin, China

**Keywords:** Cancer therapeutic resistance, Post-translational modifications, Non-small-cell lung cancer

## Abstract

Lung squamous cell carcinoma (LUSC) is associated with high mortality and has few therapeutic options. Chemotherapy remains the main treatment for LUSC patients, but multi-drug resistance has become the dominant challenge in the failure of chemotherapy in various cancers. Therefore, the effective therapeutic strategy for LUSC patients is an urgent unmet need. Here, we found vasohibin-2 (VASH2) was a prognostic biomarker for LUSC patients, and VASH2 promoted the malignant biological behaviors of LUSC cells and chemoresistance by increasing the detyrosination of α-tubulin. The high level of detyrosinated-tubulin was negatively associated with patient prognosis. Blocking the tubulin carboxypeptidase (TCP) activity of VASH2 inhibited the xenograft tumor growth and improved the treatment efficacy of paclitaxel in vivo. Results revealed that VASH2-induced increase in tubulin detyrosination boosted the binding of kinesin family member 3C (KIF3C) to microtubules and enhanced KIF3C-dependent endosomal recycling of EGFR, leading to the prolonged activation of PI3K/Akt/mTOR signaling. This study demonstrated that VASH2 was not only a prognostic biomarker but also a promising therapeutic target in LUSC, which offers a novel insight that combination of chemotherapy and EpoY, a TCP inhibitor, may be a promising treatment strategy for LUSC patients.

## Introduction

Non-small-cell lung cancer (NSCLC) is a heterogeneous disease that accounts for ~85% of lung cancer which remains a leading cause of cancer-associated mortality throughout the world [1]. Unlike lung adenocarcinoma (LUAD), patients with lung squamous cell carcinoma (LUSC) have not benefited from targeted therapies such as EGFR tyrosine-kinase inhibitors (TKIs) due to the low mutation rate [2]. Whereas EGFR overexpression is more frequently observed in LUSC than in LUAD, and aberrfant activation of EGFR plays a key role in cancer development [3, 4]. Though immune checkpoint inhibitors have revolutionized the treatment of various cancers in recent years, the low response rate and severe immune-related adverse events limit the clinical benefit in most LUSC patients [5]. Currently, chemotherapy is still the main treatment for LUSC patients, but multi-drug resistance has become the dominant challenge in the failure of chemotherapy in various cancers. Therefore, it is of vital importance to seek more therapeutic regimens to overcome multi-drug resistance and enhance the efficacy of conventional treatments for LUSC patients.

Our previous study demonstrated that vasohibin-2 (VASH2) promoted lymphatic metastasis of LUSC by facilitating lymphangiogenesis [6]. Actually, VASH2 was first identified as an angiogenic factor in endothelial cells [7]. Studies have reported that VASH2 is able to promote tumor growth by accelerating angiogenesis in various cancers, including breast cancer [8], liver cancer [9], ovarian cancer [10], and gastrointestinal cancer [11]. However, besides promoting tumor angiogenesis and lymphangiogenesis, other roles of VASH2 in tumor progression are poorly studied.

Recent studies have revealed that VASH2 exhibits tubulin carboxypeptidase (TCP) activity that is related to microtubule (MT) function [12–15]. MTs, as the major component of the cytoskeleton, are able to promote carcinogenesis by regulating intracellular trafficking of cytoplasmic components and signaling molecules after tubulin post-transitional modifications (PTMs). The most frequently occurring PTMs on MTs include detyrosination, acetylation, polyglutamylation and polyglycylation [16, 17]. Alterations in tubulin PTMs have been implicated in cancer progression [18]. Studies have reported that abnormal levels of tubulin detyrosination are associated with poor outcome and tumor aggressiveness [18–20], but the underlying mechanism remains unclear. Hence, it is important to explore the molecular mechanism of VASH2-induced tubulin detyrosination in tumor development.

In the present study, we found VASH2 was negatively associated with LUSC patient prognosis. The level of detyrosinated (deY)-tubulin was upregulated by VASH2 in LUSC cells and negatively correlated with LUSC progression and patient prognosis. Inhibiting the tubulin carboxypeptidase (TCP) activity of VASH2 attenuated the malignant biological behaviors of LUSC cells and improved the sensitivity of chemotherapeutic drugs. Importantly, we first demonstrated that the VASH2-induced increase in tubulin detyrosination boosted the binding of kinesin family member 3C (KIF3C) to MTs and promoted KIF3C-dependent endosomal recycling of EGFR, contributing to the prolonged activation of downstream PI3K/Akt/mTOR signaling. Knockdown of KIF3C rescued the VASH2-related onco-promotive function and chemoresistance. Collectively, our findings illustrate that VASH2 is not only a prognostic biomarker but also a promising therapeutic target in LUSC, which offers a novel insight that combination of chemotherapy and EpoY, a TCP inhibitor, may be a promising treatment strategy for LUSC patients.

## Materials and methods

### Patient cohorts and ethics

The clinical pathological information of enrolled LUSC patients in this study is shown in Table S1, including 119 cases of partial lung resection performed at Tianjin Cancer Hospital (TJCH cohort), 474 cases with LUSC specimen information downloaded from The Cancer Genome Atlas (TCGA cohort), and 110 LUSC tissue microarrays purchased from the Superchip Co., Ltd. (Superchip, Shanghai, China) (SupCh cohort). This project was approved by the ethics committee of Tianjin Medical University Cancer Institute and Hospital (Approval No. Ek2021143) and written informed consent was obtained from the patients. All experiments were performed in accordance with the principles of the Declaration of Helsinki.

### Cell culture

Human LUSC cell line NCI-H520 cells (Meisen, Zhejiang, China) were cultured in RPMI 1640 medium (GIBCO, NY, USA) supplemented with 10% fetal bovine serum (FBS), 100 U/ml penicillin and 100 μg/mL streptomycin (1% P/S). SK-MES-1 cells (Cellcook, Guangzhou, China) were maintained in Eagle’s Minimum Essential Medium (GIBCO, NY, USA) containing 10% FBS, 1% non-essential amino acids and 1% P/S. BEAS-2B cells (Cellcook, Guangzhou, China) were grown in Dulbecco’s Modified Eagle Medium (DMEM) with 10% FBS and 1% P/S. Human LUAD cell line A549 cells (OriCell, Guangzhou, China) were cultured in DMEM/F-12 (GIBCO, NY, USA) with 10% FBS. All cell lines were identified by short tandem repeat (STR) analysis and routinely tested for mycoplasma contamination using MycoFluor Reagent kit (Invitrogen, CA, USA). All the cells were maintained at 37˚C in a humid chamber with 5% CO_2_ and 95% air atmosphere.

### Construction of stable cell lines

For overexpression of VASH2, human full-length cDNA of VASH2 or the enzymatic inactive mutant C158A of VASH2 was subcloned into the pHBLV-CMV-MCS-3flag-EF1-puromycin lentivirus vector (Hanbio, Shanghai, China). For knockdown of VASH2 or KIF3C, target shRNA sequences (shown in Table S2) were subcloned into the pLVX-U6-CMV-RFP-P2A-BSD lentivector (Hanbio, Shanghai, China). To construct stable H520 or SK-MES-1 cell lines, cells were transduced with the corresponding lentivirus, then selected with 2 μg/mL puromycin or blasticidin for 2 weeks. The surviving cells were picked out and seeded into 96-well plates for formation of cell clones and further expansion. The VASH2-overexpressing and negative control (NC) stable cell lines were named H520^VASH2^, H520^NC^, SK^VASH2^ and SK^NC^. VASH2-C158A-overexpressing stable cells were named H520^VASH2-mut^. The stable cell lines of VASH2 knockdown and NC cells were named H520^shVASH2^ and H520^shNSC^, respectively.

The paclitaxel-resistant cell line, SK-MES-1^PTX^, was established through continuous low-dose induction combined with a concentration-escalating approach [21]. SK-MES-1^PTX^ cells were induced by treating SK-MES-1 cells with 1 nM paclitaxel initially and then increasing dose incrementally every three days, up to 20 nM finally. The parental cell line, SK-MES-1^CTRL^, was maintained in standard medium and served as the control.

### Cell viability assay

Cell Counting Kit-8 (CCK-8; UElandy, Shanghai, China) was used to detect cell viability and proliferative capacity. Cells were inoculated in 96-well plates at a density of 2 × 10^3^ cells/100 μl/well with or without treatment. After the indicated times, CCK-8 reagents were added (10 μl/well in fresh complete medium) and incubated for 2 h at 37 °C. The absorbance at 450/650 nm was measured by a microplate reader. The mean values of three replicates were calculated.

### Cell apoptosis assay

An Annexin V-FITC Apoptosis Assay Kit (Beyotime, Shanghai, China) was used to detect cell apoptosis. The cells were collected and washed once with PBS. After centrifugation at 1000 × *g* for 5 min, the cells were resuspended at a concentration of 1 × 10^5^ cells/mL to prepare a single-cell suspension. Subsequently, 5 μl of Annexin V and 10 μl of propidium iodide were added to the cell suspensions and the mixtures were incubated for 15 min at room temperature in the dark. The proportion of apoptotic cells (Annexin V+, PI−) was determined by flow cytometry.

### Wound healing assay

Wound healing assay was performed according to the protocol used in our previous study [22]. Cells were seeded in 6-well plates. When cell confluence reached about 95%, a 10-μl pipette tip was used to create a scratch on the cell monolayers. Afterward, cells were cultured in serum-free medium, and cell images were taken at 0 h and 24 h after scratch injury under an optical microscope. Wound healing assays were performed three times with three biological repetitions each time.

### Transwell assay

Transwell plates coated with Matrigel reagent (BD Biosciences, NJ, USA) were used to analyze cell invasive capacity. Cells suspended in serum-free medium were added to the upper compartments, and the lower compartments were filled with culture medium plus 10% FBS. After cultivation for 24 h, cells still remaining in the upper compartments were removed with cotton swabs, and cells which had penetrated through the membrane were fixed with methanol and stained with 0.5% crystal violet. The number of invading cells was then counted under a light microscope. Transwell assays were conducted in three independent repetitions.

### Sphere formation assay

Cells were inoculated in non-adhesive 6-well plates at a density of 1.5 × 10^3^ cells/well and cultured in DMEM/F12 medium supplemented with 10 ng/mL basic fibroblast growth factor (FGF), 20 ng/mL epidermal growth factor (EGF), and 0.4% bovine serum albumin (BSA). The number and diameter of spheres were assessed under a microscope after 10 days.

### Western blotting

Protein samples were prepared with RIPA buffer (Solarbio, Beijing, China). The concentration of protein samples was analyzed using the BCA Protein Assay Kit (Thermo Fisher Scientific, MA, USA). Protein samples (20 μg/lane) were electrophoresed on a 10% separating gel and blotted onto a polyvinylidene difluoride membranes (Millipore, MA, USA). After blocking with 5% skim milk for 1 h at room temperature, the membranes were incubated with primary antibody overnight at 4 °C, then washed three times with Tris-buffered saline-Tween buffer. Afterwards, the membranes were incubated with the appropriate secondary antibody for 1 h at room temperature. The protein bands were visualized using an enhanced chemiluminescence kit (Pierce, IL, USA), and the intensity of protein bands was analyzed using Image J analysis software. All antibodies used are shown in Table S3.

### EGFR internalization assay

The assay was performed as previously reported [23]. Briefly, cells at 90% confluence were serum-starved for 1 h in serum-free uptake media containing 20 mM HEPES. The cells were then incubated with 2 μg/mL of Alexa Fluor 647-labelled EGF (Thermo Fisher Scientific, MA, USA) in ice water for 30 min. After washing with ice-cold PBS, cells were incubated for 0, 5 and 10 min at 37 °C to allow internalization. The cells were placed on ice to stop internalization and then subjected to an acidic wash (0.2 M sodium acetate, pH 3.5) for 2 min to remove non-internalized fluorescent-conjugated EGF. Cells were collected and the fluorescence emission of internalized EGF was detected by flow cytometry.

### Biotinylation and recycling assay of EGFR

Measurement of EGFR recycling has been described previously [24]. Briefly, cells were surface-labeled with 0.5 mg/mL EZ-Link Sulfo-NHS-SS-Biotin (Thermo Scientific, MA, USA) on ice for 30 min. The reactions were quenched with 50 mM Tris on ice for 15 min. Cells were then incubated in serum-free medium with 20 ng/mL EGF (SinoBiological, Beijing, China) for 30 min at 37 °C to allow internalization of tracer into the perinuclear recycling compartment. Biotin remaining at the cell surface was removed by exposure to MesNa (50 mM Tris, 100 mM NaCl, 1 mM EDTA, pH 8.6, 0.2% BSA) three times (4 °C for 10 min). After, the cells were re-incubated at 37 °C in serum-free medium for the indicated times to allow recycling of the internalized EGFR. Cells were then re-exposed to MesNa three times (4 °C for 10 min) to remove the biotin conjugated to membrane EGFR. After washing with ice-cold PBS, the cells were lysed and the biotinylated EGFR was quantified by immunoprecipitation with streptavidin-agarose beads, followed by western blotting. Recycling % was calculated using the formula: Recycling % = (total − remaining)/total × 100%.

### EGF-induced EGFR degradation

Cells at 90% confluence were treated with 50 µg/mL cycloheximide (MedChemExpress, NJ, USA) in serum-free medium for 2 h to block de novo synthesis of EGFR. A total of 20 ng/mL EGF (SinoBiological, Beijing, China) was added to the cells and incubated for 0, 1, 3, and 5 h at 37 °C. The incubation was stopped by placing the plates on ice and washing three times with ice-cold PBS. Cell lysates were collected and the amount of EGFR was measured by western blotting.

### MT co-sedimentation

The assay was conducted as previously reported [25]. In brief, cells were lysed in solubilizing buffer (80 mM PIPES, 1 mM EGTA, 1 mM MgCl2, pH 6.9) supplemented with 1 mM GTP, 1 mM DTT, 1% NP40 and protease inhibitor. After incubation on ice for 30 min, the lysates were centrifuged at 200,000 × *g* for 30 min at 4 °C to remove nuclei and cellular debris. The supernatant was divided into three tubes, with one serving as input, and the other tubes receiving taxol (2 μM) or nocoodazole (1 μM). After incubation for 30 min at 37 °C (taxol) or 30 min at 4 °C (nocodazole), the reaction mixture was centrifuged at 100000 × g for 30 min at room temperature through a 10% sucrose cushion. Cell lysates were fractionated to separate the co-sediment containing the MT polymer and the supernatant containing monomer/dimer tubulins. Each fraction was analyzed by western blotting.

### Immunofluorescence (IF) assay

Cells growing on coverslips were washed with PBS, fixed in 4% paraformaldehyde for 15 min and treated with 0.1% Triton-X-100 in PBS for 5 min. Cells were blocked with 5% BSA for 1 h at room temperature and incubated with specific primary antibodies and then the corresponding dye-conjugated secondary antibody. All antibodies used are shown in Table S3. Finally, cells were counterstained with DAPI (Solarbio, Beijing, China). Fluorescent images were acquired using a Zeiss LSM880 confocal microscope.

### Co-immunoprecipitation (co-IP)

The Protein A/G Magnetic Beads Immunoprecipitation (IP) Kit (SinoBiological, Beijing, China) was used to immunoprecipitate equal amounts of target protein with the primary antibody. All antibodies used are shown in Table S3. All procedures were performed following the manufacturer’s instructions, and immunoprecipitation with rabbit IgG was also performed as a negative control. The final supernatants were collected for further western blotting assay, with 1% of the crude lysates used as input.

### Immunohistochemistry (IHC) assay

IHC was performed to measure the expression of target protein in tissues according to our previous study [26]. The antibodies we used here are shown in Table S3 according to the manufacturer’s information. Immunohistochemistry staining was assessed by two independent pathologists with no prior knowledge of patient characteristics. Discrepancies were resolved by consensus. The staining extent score of target protein, from 0 to 3, corresponds to the percentage of immunoreactive tumor cells (0%, 1%–25%, 26%–75%, and 76%–100%, respectively). The staining intensity was scored as negative (score = 0), weakly positive (score = 1), positive (score = 2), or strongly positive (score = 3).

### Xenograft tumor model

BALB/c female nude mice (female, 4-6 weeks old, purchased from Beijing Sibeifu Animal Technology Co.) were acclimated for 5 days and were selected randomly to subcutaneously (s.c.) injected with 100 μl suspensions of 5 × 10^6^ H520 cells overexpressing VASH2. All mice were housed in animal facility under specific pathogen-free conditions. When tumor volumes reached about 100 mm^3^, the nude mice were randomly divided into three groups. The tumor volume and body weight were measured every two days. Tumor dimension was analyzed as length × width^2^ × 0.5. For the therapy models, 10 mg/kg paclitaxel (TargetMol, MA, USA) or 10 mg/kg EpoY (Merck Millipore, MA, USA) or PBS was intraperitoneally injected into the mice three alternate days weekly. At 2–3 weeks, the nude mice were sacrificed by the CO_2_ asphyxia method, and tumors were weighed. In accordance with the ethical guidelines, the tumor volume did not reach 1500 mm^3^ or ulcers happened.

All animal experiments complied with the Animal Research: Reporting of In Vivo Experiments (ARRIVE) guidelines, and were performed in accordance with the National Institutes of Health guidelines for the care and use of Laboratory animals (NIH Publications No. 8023, revised 1978). The animal experiments were approved by the Animal Ethical Committee of Tianjin Medical University Cancer Institute and Hospital (AE-2023016). Furthermore, there were no blinding study for different animal groups.

### Statistical analysis

All statistical calculations were performed using GraphPad Prism (version 8.0). Unless otherwise stated, the data are presented as the mean ± SD of at least three independent experiments. Student’s t-tests (two-tailed) were applied to assess the statistical significance of differences between any two groups. Multiple groups were compared by using one-way analysis of variance. Chi-square tests were performed to assess clinical and biological variable differences between patient groups. Survival analysis was carried out using the Kaplan–Meier method for estimation of event rates and the log-rank test for survival comparisons between patient groups. Differences with p-values less than 0.05 were considered statistically significant.

## Results

### VASH2 increased the detyrosination of α-tubulin and promoted the malignant biological behaviors of LUSC cells

**Fig. 1:**
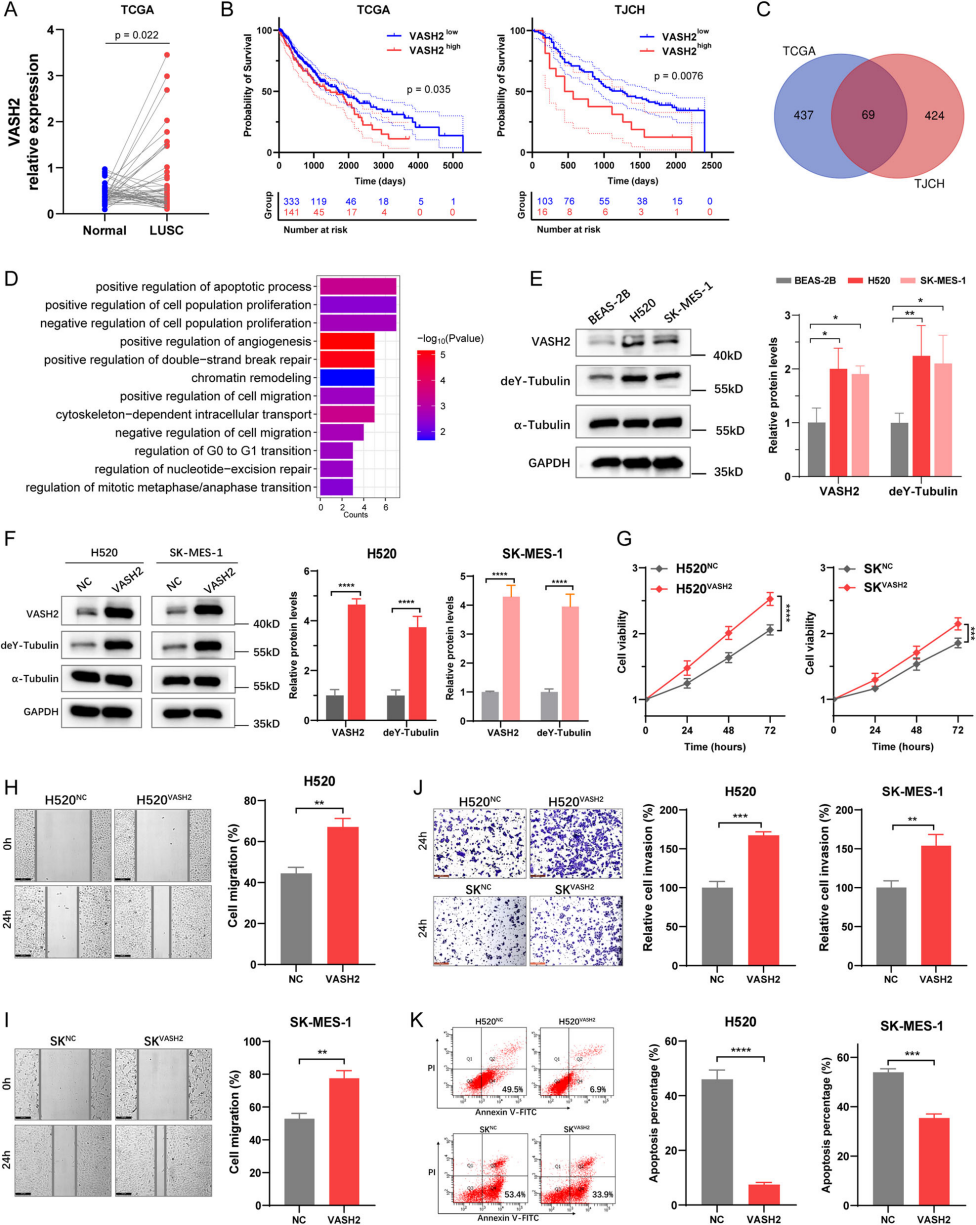
VASH2 increased the detyrosination of α-tubulin and promoted malignant biological behaviors of LUSC cells. **A** VASH2 was upregulated in LUSC tissues compared to the para-carcinoma tissues based on the TCGA datasets. **B** Kaplan–Meier curves representing overall survival of LUSC patients stratified based on VASH2 expression in TCGA cohort (left) and TJCH cohort (right). **C** Venn diagram of VASH2-related differential genes in both TCGA and TJCH cohorts. **D** GO pathway enrichment analysis of the intersecting VASH2-related differential genes in TCGA and TJCH cohorts. **E** Representative images of western blotting and quantification for the expression of VASH2, deY-tubulin, and α-tubulin in BEAS-2B, H520 and SK-MES-1 cells. GAPDH was used as an internal control. **F** Representative images of western blotting and quantification for the expression of VASH2, deY-tubulin and α-tubulin after the overexpression of VASH2 in H520 and SK-MES-1 cells. GAPDH was used as an internal control. **G** Cell proliferation of H520^VASH2^ and SK^VASH2^ cells was increased compared to the negative control cells, determined by CCK-8 assays. **H**–**I** Representative images (left) and cell migration quantification (right) of wound healing assays after overexpression of VASH2 in H520 cells (**H**) and SK-MES-1 cells (**I**). Scale bar, 200 μm. **J** Representative images (left) and cell invasion quantification (right) of trans-well assays after overexpression of VASH2 in H520 and SK-MES-1 cells. Scale bar, 200 μm. **K** Cell apoptosis of H520^VASH2^ and SK^VASH2^ cells was decreased compared to the negative control cells, determined by Annexin V-FITC assays. Data were representative of three independent experiments. The student’s t tests, *, *p* < 0.05; **, *p* < 0.01; ***, *p* < 0.001 and ****, *p* < 0.0001.

We found that VASH2 expression was upregulated in LUSC tissues compared to the para-carcinoma tissues (Fig. 1A), and was negatively correlated with LUSC patient prognosis (Fig. 1B). To investigate the role of VASH2 in LUSC, we conducted a differential gene expression analysis in both the TCGA and TJCH cohorts. A Venn diagram identified 69 intersecting VASH2-associated differentially expressed genes between the two cohorts (Fig. 1C). GO pathway enrichment analysis of these 69 common genes suggests that VASH2 may play a role in the regulation of apoptosis, cell proliferation, angiogenesis, cell migration, and cytoskeleton-dependent vesicle transport (Fig. 1D). Recent studies have reported that VASH2 is a carboxypeptidase that can mediate detyrosination of α-tubulin [14, 15], but little is known about the role of VASH2-induced tubulin detyrosination in tumorigenesis. Therefore, we next conduct functional validation through cell experiments.

We first assessed VASH2 expression in various LUSC cell lines (H520 and SK-MES-1) as well as in a normal lung epithelial cell line (BEAS-2B). Western blotting analysis revealed that both VASH2 and deY-tubulin levels were nearly two-fold higher in LUSC cells compared to BEAS-2B cells (Fig. 1E). Additionally, VASH2 overexpression significantly increased deY-tubulin levels in both H520 and SK-MES-1 cells compared to negative control cells, with the differences being statistically significant (Fig. 1F). Meanwhile, cell proliferation (Fig. 1G), migration (Fig. 1H, I) and invasion (Fig. 1J) were all increased in H520^VASH2^ and SK^VASH2^ cells compared to negative control cells. While, apoptosis was significantly reduced in both H520^VASH2^ and SK^VASH2^ cells (Fig. 1K) relative to the controls. In addition, knockdown of VASH2 resulted in a decreased level of deY-tubulin in H520 cells (Fig. S1A). Furthermore, VASH2 silencing significantly attenuated cell proliferation (Fig. S1B), migration (Fig. S1C), and invasion (Fig. S1D), while promoting apoptosis (Fig. S1E) compared to control cells. The sphere-forming ability of H520 cells was also diminished after VASH2 knockdown (Fig. S1F).

More importantly, the increased level of deY-tubulin caused by VASH2 overexpression was remarkably reduced (Fig. S1G) by treatment with EpoY, a TCP inhibitor. Accordingly, the cell proliferation, migration and invasion of H520^VASH2^ cells were all decreased after treatment with EpoY (Fig. S1H–J). Taken together, our data demonstrated that VASH2 facilitated the malignant biological behaviors of LUSC cells by increasing detyrosination of α-tubulin.

### Increased level of deY-tubulin was negatively associated with patient prognosis and facilitated LUSC progression

**Fig. 2:**
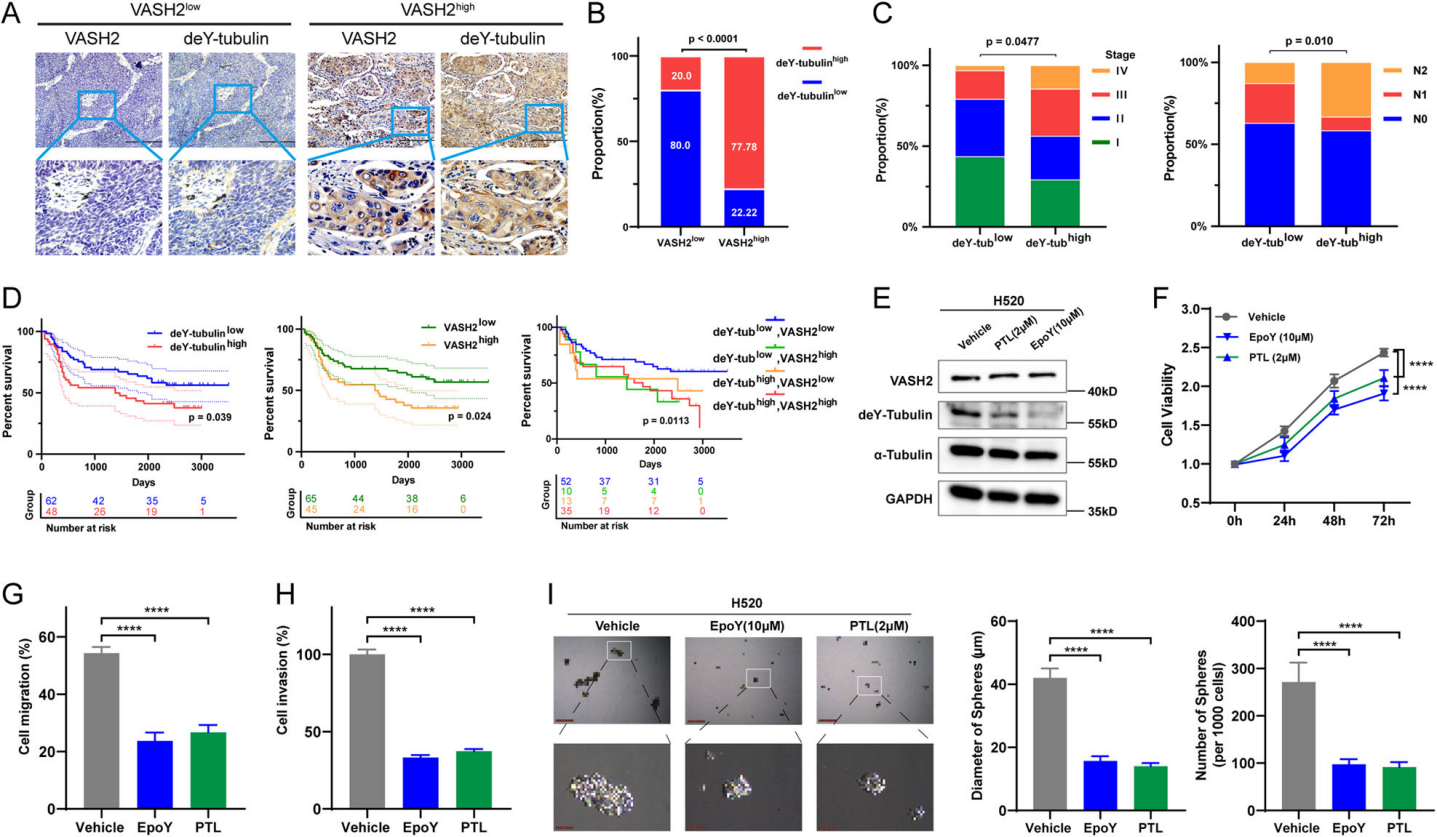
Increased level of detyrosinated-tubulin was negatively associated with patient prognosis and facilitated LUSC progression. **A** Representative images of deY-tubulin immunohistochemical staining in VASH2^low^ and VASH2^high^ LUSC tissues from the SupCh cohort. Scale bar, 100 μm. **B** The level of deY-tubulin was positively correlated with VASH2 protein in LUSC tissues from the SupCh cohort (*p* < 0.0001). *p* values were calculated by chi-square test. **C** High level of deY-tubulin was associated with advanced clinical stages (left) and more lymph node metastasis (right). *p* values were calculated by log-rank test. **D** Overall survival analysis of LUSC patients in the SupCh cohort stratified according to VASH2 and deY-tubulin levels. *p* values were calculated by log-rank test. **E** Levels of VASH2 and deY-tubulin were detected by western blotting 24 hours after addition of two TCP inhibitors, EpoY (10 µM) or PTL (2 µM), to H520 cells. GAPDH was used as an internal control. **F** H520 cell proliferation was decreased by both TCP inhibitors. **G** Migration of H520 cells was assessed 24 hours after addition of EpoY (10 µM) or PTL (2 µM). **H** The invasion ability of H520 cells was determined 24 hours after of treatment with EpoY (10 µM) or PTL (2 µM). **I** Representative images of H520 sphere formation assays after addition of two TCP inhibitors (left) and quantification based on diameter and number of spheres (right). Data were representative of three independent experiments. The One-Way ANOVA tests, *, *p* < 0.05; **, *p* < 0.01; ***, *p* < 0.001 and ****, *p* < 0.0001.

To explore the role of tubulin detyrosination in LUSC, we detected the deY-tubulin levels in LUSC tissues from the SupCh cohort by IHC assays (Fig. 2A). Results indicated that deY-tubulin had a higher level in the VASH2^high^ group than in the VASH2^low^ group (*p* < 0.001; Fig. 2B). A comparison of clinical pathological profiles of patients with varying deY-tubulin and VASH2 expression was shown in Table S4, which indicated that high levels of deY-tubulin were associated with advanced clinical stages and increased lymph node metastasis (Fig. 2C). LUSC patients with high levels of deY-tubulin or VASH2 exhibited poorer prognoses, while those with low expression of both VASH2 and deY-tubulin demonstrated the best overall survival (Fig. 2D). These results indicate that VASH2-related increase in tubulin detyrosination may contribute to the progression of LUSC.

To further validation, we applied two inhibitors in LUSC cells, EpoY [15] and parthenolide [27], to reduce the detyrosination of tubulin. Western blotting results showed that both of the inhibitors decreased the levels of deY-tubulin without affecting the levels of VASH2 protein in LUSC cells (Fig. 2E). Additionally, these inhibitors effectively attenuated LUSC cell proliferation (Fig. 2F), migration (Fig. 2G), and invasion (Fig. 2H). Moreover, the sphere-forming ability of H520 cells was significantly decreased in both diameter and number (Fig. 2I). Collectively, high level of tubulin detyrosination may promote LUSC cell proliferation, migration and invasion, as well as cell stemness.

### EpoY abolished VASH2-induced chemoresistance and xenograft tumor growth

**Fig. 3:**
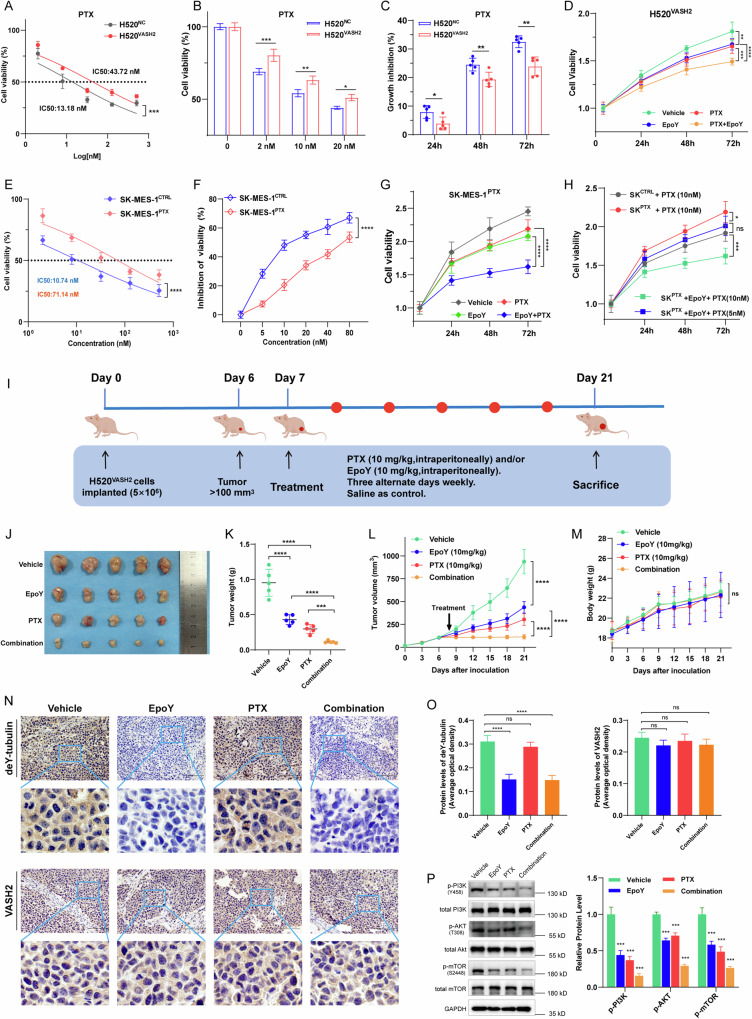
EpoY abolished VASH2-induced chemoresistance and xenograft tumor growth. **A** The IC_50_ values of paclitaxel (PTX) in H520^NC^ and H520^VASH2^ cells was determined 48 h post-treatment. **B** Cell viability was measured by treatment with 2 nM, 10 nM, and 20 nM PTX in H520^NC^ and H520^VASH2^ cells for 48 h. **C** H520^NC^ and H520^VASH2^ cells were incubated with PTX (2 nM) for 24 h, 48 h and 72 h. Cell viability was evaluated by CCK-8 assay. **D** Anticancer activity of PTX (2 nM), EpoY (10 μM), and PTX-EpoY combination against H520^VASH2^ cells. **E** The IC_50_ values of PTX in SK-MES-1^CTRL^ and SK-MES-1^PTX^ cells was determined 48 h post-treatment. **F** Cell viability was measured by treatment with different concentration of PTX in SK-MES-1^CTRL^ and SK-MES-1^PTX^ cells for 48 h. **G** Anticancer activity of PTX (10 nM), EpoY (50 µM) and PTX-EpoY combination against SK-MES-1^PTX^ cells. **H** Comparing the anticancer activity of PTX alone in SK-MES-1^CTRL^ cells and PTX-EpoY combinations in SK-MES-1^PTX^ cells. **I** Experimental design for PTX and EpoY treatment in the H520^VASH2^-xenograft mouse model (five mice per group). **J**–**L** General view (**J**), tumor weight (**K**) and tumor volume (**L**) of xenograft tumors with different treatment. **M** The curve of mice body weight. **N**, **O** Representative images (**N**) and quantification (**O**) of deY-tubulin and VASH2 IHC staining in xenograft tumors treated with PTX and/or EpoY. Scale bar, 100 μm. **P** Western blotting (left) and quantification (right) of the phosphorylation of PI3K/Akt/mTOR in xenograft tumors. Groups compared using one-way ANOVA analysis, *, *p* < 0.05; **, *p* < 0.01; ***, *p* < 0.001 and ****, *p* < 0.0001.

Chemotherapy remains the main treatment for LUSC patients. Unfortunately, multi-drug resistance has become the dominant challenge in the failure of chemotherapy in various cancers [28, 29]. To investigate whether the TCP activity of VASH2 had an impact on chemoresistance in LUSC, we chose several first-line chemotherapeutic drugs for LUSC, including paclitaxel, cisplatin and gemcitabine. Results showed that the half-maximal inhibitory concentration (IC_50_) of paclitaxel, cisplatin and gemcitabine were all increased in H520^VASH2^ cells compared to H520^NC^ cells (Figs. 3A and S2A). Then we treated H520^NC^ and H520^VASH2^ cells with paclitaxel at concentrations of 2 nM, 10 nM, and 20 nM, assessing cell viability after 48 hours to determine the optimal concentration. The results indicated a dose-dependent decrease in cell viability in both cell lines. While, as the concentration of paclitaxel increased, the difference in cell viability between H520^NC^ and H520^VASH2^ cells gradually narrowed, with the most significant difference observed at 2 nM paclitaxel (*p* = 0.0003, Fig. 3B). The use of a lower concentration of paclitaxel minimized cytotoxicity while allowing cells to retain certain proliferative viability to evaluate differential drug responses. Therefore, we selected the concentration of 2 nM to verify the inhibitory effect of paclitaxel on cell proliferation, and the results showed that 2 nM paclitaxel significantly inhibited the proliferation of H520^NC^ cells more effectively than that of H520^VASH2^ cells (Fig. 3C).

Additionally, we employed a similar approach to evaluate the dose-response effect of cisplatin and gemcitabine, determining the optimal concentrations for each. The results showed that when using 1 μM of either cisplatin or gemcitabine, the difference in cell viability between H520^NC^ and H520^VASH2^ cells was most significant, outperforming the effects observed at higher concentrations (Fig. S2B). Furthermore, both 1 μM cisplatin and 1 μM gemcitabine significantly inhibited the proliferation of H520^NC^ cells more effectively than that of H520^VASH2^ cells (Fig. S2C). These results demonstrated that high level of VASH2 decreased the sensitivity of LUSC cells to chemotherapeutic drugs, including paclitaxel, cisplatin and gemcitabine. Notably, the sensitivity of H520^VASH2^ cells to paclitaxel, cisplatin and gemcitabine was increased by combined treatment with EpoY (Figs. 3D and S2D), suggesting that inhibiting the TCP activity of VASH2 could restore the reduced chemotherapy sensitivity associated with VASH2.

To verify the potential of the TCP inhibitor in overcoming chemotherapy resistance, we established a paclitaxel-resistant cell line, SK-MES-1^PTX^, through continuous low-dose induction combined with a concentration-escalating approach [21]. Compared to the parental cell line SK-MES-1^CTRL^, the IC_50_ of SK-MES-1^PTX^ cells increased from 10.74 nM to 71.14 nM (*p* < 0.0001, Fig. 3E). The inhibitory effect of paclitaxel on the viability and proliferation of SK-MES-1^PTX^ cells was significantly lower than on SK-MES-1^CTRL^ cells (Figs. 3F and S6A). Furthermore, the combined use of EpoY and paclitaxel at a 5:1 mass ratio significantly reduced the cell viability compared to paclitaxel alone, with a remarkable decrease at a paclitaxel concentration of 10 nM (Fig. S6B). Moreover, 50 µM EpoY combined with 10 nM paclitaxel exhibited significantly superior anticancer activity against SK-MES-1^PTX^ cells compared to either agent alone (Fig. 3G). Notably, compared to the efficacy of 10 nM paclitaxel alone in SK-MES-1^CTRL^ cells, the combination with EpoY effectively overcame paclitaxel resistance in SK-MES-1^PTX^ cells. Importantly, EpoY could even be used in combination with a much lower concentration of paclitaxel (5 nM) to achieve the efficacy of 10 nM paclitaxel alone in SK-MES-1^CTRL^ cells (Fig. 3H).

To define the combinatorial effect of paclitaxel and EpoY as additive or synergistic, the combination index (CI) value of paclitaxel-EpoY combination was analyzed using Compusyn software [30, 31]. Results showed that paclitaxel-EpoY combination had a synergistic effect against paclitaxel-resistant SK-MES-1^PTX^ cells with the CI values ranging from 0.362–0.993 for the fraction affected by the dose (Fa) from 5–97% (Fig. S6C). Altogether, these results suggest that the synergistic effect of EpoY and paclitaxel could overcome the resistance of LUSC cells to paclitaxel and allowed for a reduction in the dosage of paclitaxel, which would help to mitigate the cytotoxicity associated with paclitaxel.

To further evaluate the efficacy of the combined treatment with paclitaxel and the TCP inhibitor against VASH2-induced paclitaxel resistance in vivo, we established a BALB/c-nu xenograft model using H520^VASH2^ cells and analyzed the tumor control rate (Fig. 3I). Both paclitaxel and EpoY could inhibit the growth of xenograft tumors (Fig. 3J, K). Importantly, compared to paclitaxel alone, combined treatment increased the inhibition of tumor growth by 20.43% (*p* = 0.0003; Fig. 3L). Mouse growth was not affected by single-agent or combined treatment (Fig. 3M), and no severe toxicities were observed in mouse liver, kidney or lung as shown by hematoxylin-eosin staining (Fig. S2E), which indicated that EpoY is not significantly toxic in vivo. Immunohistochemistry of tumor sections showed that the use of EpoY decreased the level of deY-tubulin without affecting the expression of VASH2 (Fig. 3N, O). Besides, inhibiting the TCP activity of VASH2 reduced both the proportion of proliferative Ki67+ cells and microvessel density of CD31 compared to the control group (Fig. S2F and S2G). Studies have reported that multi-drug resistance of chemotherapy often accompanies activation of PI3K/Akt pathway in various cancers [29, 32–34], thus we determined the phosphorylation levels of PI3K/Akt/mTOR pathway in xenograft tumor lysates. Results showed that the use of EpoY could enhance the inhibition of PI3K, Akt and mTOR phosphorylation (Fig. 3P). Altogether, our data demonstrated that blocking the TCP activity of VAH2 attenuated xenograft tumor growth, and paclitaxel treatment efficacy on LUSC was significantly improved in combination with EpoY in vivo.

### VASH2-induced detyrosination of α-tubulin promoted EGFR-endosomal recycling and prolonged downstream PI3K/Akt/mTOR activation

**Fig. 4:**
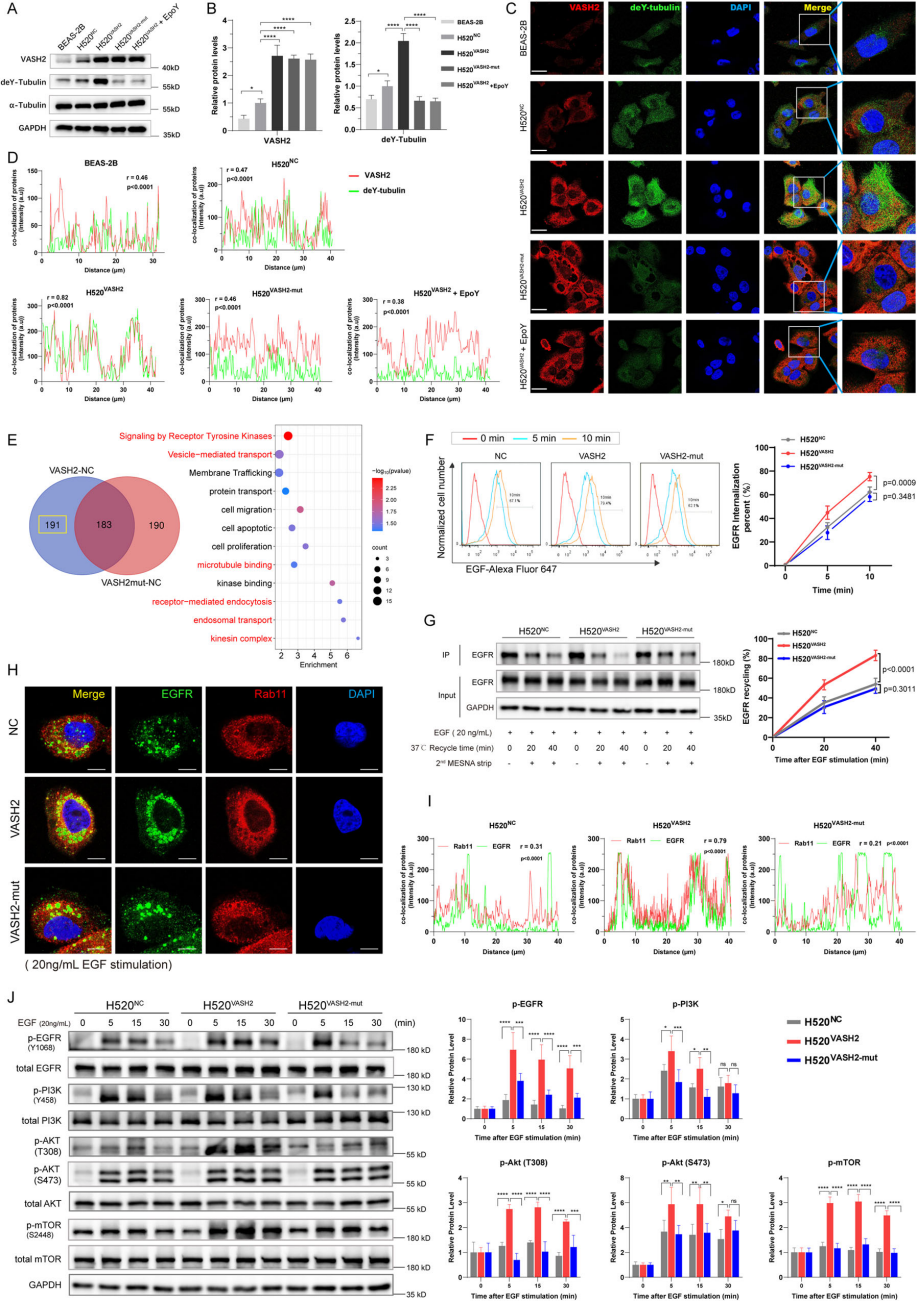
VASH2 promoted EGFR-endosomal recycling and prolonged downstream PI3K/Akt/mTOR activation by raising α-tubulin detyrosination. **A**, **B** Representative images of western blotting and quantification for the expression of VASH2, deY-tubulin in BEAS-2B, H520^NC^, H520^VASH2^, H520^VASH2-mut^, and H520^VASH2^ cells treated with 10 µM EpoY for 24 h. GAPDH was used as an internal control. **C** Representative images of immunofluorescence (IF) assays for the levels of VASH2 and deY-tubulin in BEAS-2B, H520^NC^, H520^VASH2^, H520^VASH2-mut^, and H520^VASH2^ cells treated with 10 µM EpoY for 24 h. **D** The co-localization analysis of VASH2 and deY-tubulin in the IF assays. Scale bar, 20 μm. **E** Venn diagram (left) and pathway enrichment analysis (right) of VASH2-related differentially expressed genes between H520^NC^, H520^VASH2^ and H520^VASH2-mut^ cells. **F** EGFR internalization assay using AF647-labelled EGF (2 μg/mL), and the fluorescence emission of internalized EGF-EGFR complex was detected by flow cytometry. **G** Recycling assays of EGFR in the indicated cells were conducted by using EZ-Link Sulfo-NHS-SS-Biotin (0.5 mg/mL) to label cell surface, and the biotinylated EGFR were quantified by immunoprecipitation with streptavidin-agarose beads followed by western blotting. **H**, **I** Representative images of IF assays (**H**) and the co-localization analysis (**I**) of EGFR and Rab11 in the indicated cells. Scale bar, 10 μm. **J** Western blotting (left) and quantification (right) for the levels of phosphorylated-EGFR and downstream PI3K/Akt/mTOR after 20 ng/mL EGF stimulation for 0 min, 5 min, 15 min and 30 min in the indicated cells. Data were representative of three independent experiments. Groups compared using one-way ANOVA analysis, *, *p* < 0.05; **, *p* < 0.01; ***, *p* < 0.001 and ****, *p* < 0.0001.

To elucidate the mechanism of VASH2-induced tubulin detyrosination in LUSC tumorigenesis, we constructed a VASH2 mutant stable cell line of H520 that harbored the enzymatic inactive mutant C158A of VASH2 [35]. The validation was performed by measuring the levels of VASH2 and deY-tubulin in H520^NC^, H520^VASH2^, and H520^VASH2-mut^ cells, with BEAS-2B cells as a negative control and H520^VASH2^ cells treated with 10 µM EpoY for 24 hours as a positive control. Western blotting results indicated that the deY-tubulin levels were significantly reduced in H520^VASH2-mut^ cells compared to H520^VASH2^ cells, with no significant difference between H520^VASH2-mut^ cells and H520^VASH2^ cells treated with EpoY (Fig. 4A, B). Immunofluorescence assay showed that only H520^VASH2^ cells exhibited strong bundled structures of detyrosinated microtubules and significantly enhanced the co-localization of deY-tubulin with VASH2 compared to the other groups (Fig. 4C, D).

To explore the underlying molecular basis for TCP activity of VASH2 in LUSC, we conducted the global gene expression profiling analysis using whole genomic transcriptional sequencing among stable cell lines H520^NC^, H520^VASH2^ and H520^VASH2-mut^. The Venn diagram showed that there were 191 differentially expressed genes that only showed in H520^VASH2^ cells compared to H520^VASH2-mut^ cells (Fig. 4E). Pathway enrichment analysis indicated that receptor tyrosine kinases, receptor-mediated endocytosis, vesicle-mediated transport, microtubule binding and endosomal transport pathways were involved, which were strongly associated with microtubule functions (Fig. 4E). Since microtubule play a critical role in regulating EGFR endocytosis [36], we speculated that VASH2 maybe have an impact on the EGFR endocytosis process, which involves EGFR internalization, recycling and degradation [37].

Therefore, we firstly applied flowcytometry analysis of EGFR internalization among H520^NC^, H520^VASH2^ and H520^VASH2-mut^ using AF647-labeled EGF. Results showed that the EGF internalization rate was increased in H520^VASH2^ cells compared with H520^NC^ cells (*p* = 0.0009), while there was no significant difference (*p* = 0.3481) between H520^VASH2-mut^ cells and control cells (Fig. 4F). Then, EGFR recycling was determined by cell-surface biotinylation assays. Results indicated that EGFR recycled at a faster rate in H520^VASH2^ cells than that in H520^NC^ cells (*p* < 0.0001), while H520^VASH2-mut^ cells were unable to accelerate endosomal recycling of EGFR compared to the control cells (*p* = 0.3011; Fig. 4G). Moreover, immunofluorescence assays showed the amount of EGFR co-localized with Rab11, a marker of recycling endosomes, was only increased in H520^VASH2^ cells (Fig. 4H, I). These results indicated that VASH2-induced tubulin detyrosination play a vital role in EGFR endocytosis and promoted the EGFR endosomal recycling.

Besides, we further measured EGFR degradation in H520 stable cell lines, which were stimulated with EGF (20 ng/mL) and incubated with cycloheximide (50 µg/mL) to inhibit de novo protein synthesis. Western blotting analysis indicated that EGFR degradation rates had no statistical difference between H520^VASH2^ and H520^NC^ cells (*p* = 0.552, Fig. S3A). Moreover, there was no significant difference in the co-localization of EGFR and Rab7, a marker of late endosomes, between H520^VASH2^ and control cells (Fig. S3B). These data suggested that VASH2 had little influence on early-to-late EGFR endosomal maturation and EGFR degradation.

Increased EGFR recycling is predicted to prolong signaling and plays a key role in cancer development [36, 38]. Hence, the effect of VASH2-induced tubulin detyrosination on EGFR signal transduction was detected by EGF stimulation for 0, 5, 15 and 30 min. Western blotting analysis showed that a rapid and sustained phosphorylation of EGFR along with downstream PI3K/Akt/mTOR occurred in H520^VASH2^ cells compared to H520^NC^ cells, which was not observed in H520^VASH2-mut^ cells (Fig. 4J). However, there was no significant difference in activation of the EGFR-downstream canonical mitogen-activated protein kinase (MAPK) pathway between H520^VASH2^ and H520^VASH2-mut^ cells (Fig. S3C). These results illustrated that VASH2-induced detyrosination of α-tubulin sustained the activation of downstream PI3K/Akt/mTOR signaling, but had little impact on MEK/ERK or p38 signaling.

### VASH2-induced increase in tubulin detyrosination boosted the binding of KIF3C to MTs

**Fig. 5:**
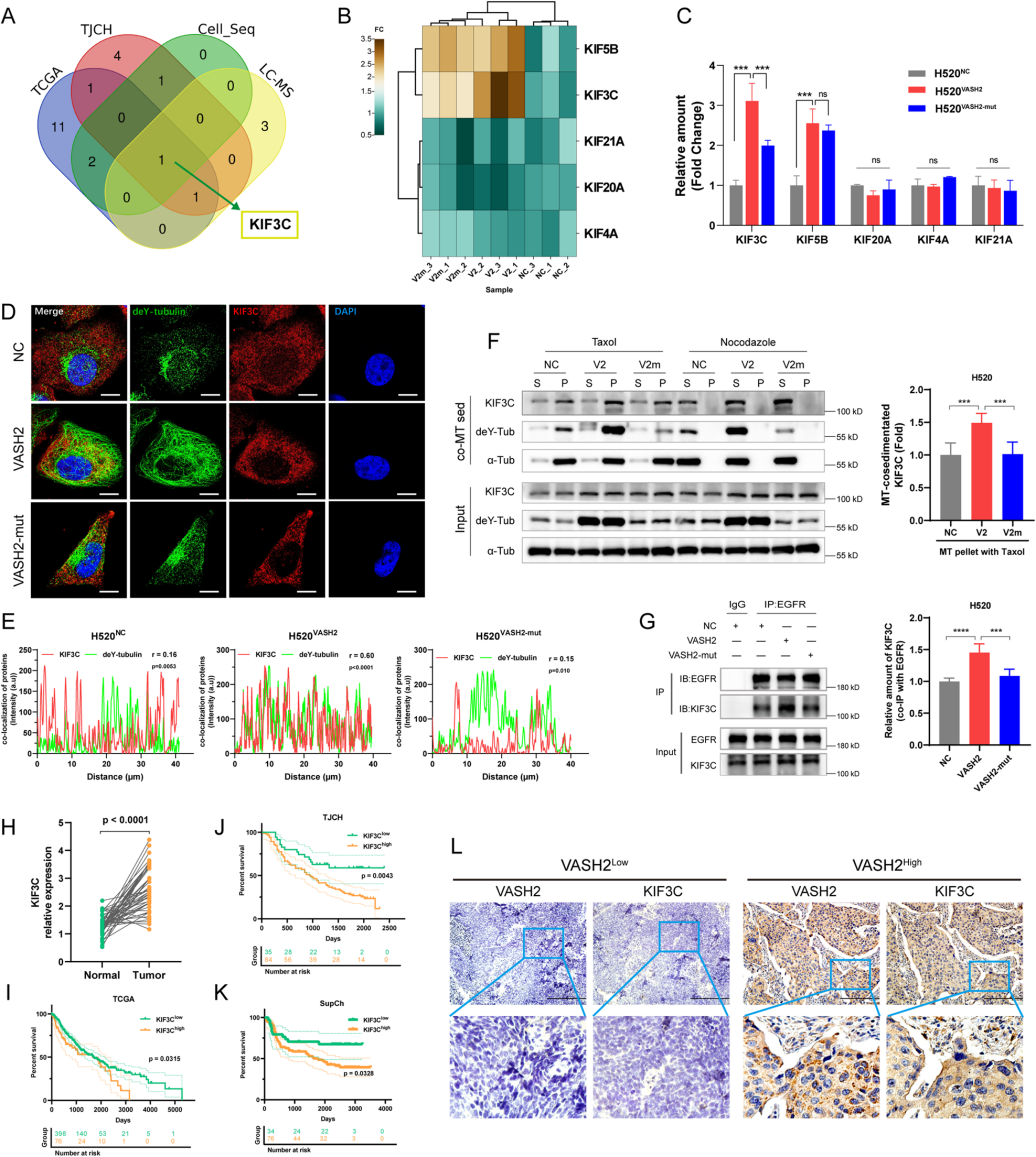
VASH2-induced increase in α-tubulin detyrosination boosted the binding of KIF3C to MTs. **A** Venn diagram showed the overlap of differentially expressed *KIF* genes between different groups, including LC-MS/MS analysis and RNA-seq of H520^VASH2^ cells, as well as LUSC tissue-sequencing from TCGA cohort and TJCH cohort. **B** The heatmap displayed the KIF proteins that bind with α-tubulin in H520^NC^, H520^VASH2^ and H520^VASH2-mut^ cells, which was detected by immunoprecipitation and LC-MS/MS. **C** Quantification of KIF proteins binding with α-tubulin in the indicated cells. **D**, **E** Representative images of IF assays (**D**) and the co-localization analysis (**E**) of KIF3C and deY-tubulin in the indicated cells. Scale bar, 10 μm. **F** The binding of KIF3C to MTs was elevated in H520^VASH2^ cells determined by MT-co-sedimentation assay. S, supernatant; P, pellet. **G** Co-IP assay of EGFR and KIF3C after 20 ng/mL EGF stimulation (left), the amount of KIF3C binding with EGFR was increased in H520^VASH2^ cells (right). **H** KIF3C expression was increased in LUSC tissues compared to the paired adjacent tissues, based on TCGA database. **I**–**K** Kaplan–Meier curves representing the overall survival of LUSC patients with low/high KIF3C expression of TCGA cohort, TJCH cohort and SupCh cohort. **L** Representative images of KIF3C immunohistochemical staining in LUSC tissues from the SupCh cohort. Scale bar, 100 μm. Data were representative of three independent experiment. The One-Way ANOVA tests, *, *p* < 0.05; **, *p* < 0.01; ***, *p* < 0.001 and ****, *p* < 0.0001.

Kinesin (KIF) proteins are the key motor proteins in MT-regulated intracellular transport of recycling endosomes and vesicles [39–42]. To figure out the key KIFs involved in VASH2-enhanced EGFR endosomal recycling, we firstly screened the differentially expressed KIFs between H520^VASH2^ and H520^NC^ cells using LC-MS/MS or RNA-seq analysis, as well as those differentially expressed KIFs between VASH2^high^ and VASH2^low^ LUSC tissues either from TCGA cohort or TJCH cohort using RNA-seq analysis (Fig. S4A). As shown in the Venn diagram, there were a total of six KIFs which intersected between groups, while KIF3C, a member of the kinesin-2 family, was the one shared among the different groups (Fig. 5A). LC-MS/MS analysis, using the samples of α-tubulin-immunoprecipitation from H520^VASH2^, H520^VASH2-mut^ and H520^NC^ cells, showed there were five KIF proteins detectable, including KIF5B, KIF3C, KIF21A, KIF20A and KIF4A (Fig. 5B). Among the five detectable KIF proteins, both KIF3C and KIF5B increased in H520^VASH2^ cells compared to H520^NC^ cells, but only the amount of KIF3C protein significantly decreased in H520^VASH2-mut^ compared to H520^VASH2^ cells, which indicated that the level of KIF3C binding with α-tubulin was affected by interfering VASH2-induced tubulin detyrosination (Fig. 5C).

Studies have demonstrated that tubulin PTMs affect the affinity, velocity and processivity of motor proteins [43, 44]. In this study, immunofluorescence assays showed that VASH2 evoked an enhancement of the radial deY-MTs networks and promoted KIF3C recruitment to deY-MTs in H520^VASH2^ cells compared to H520^NC^ and H520^VASH2-mut^ cells (Fig. 5D, E). Furthermore, MT-co-sedimentation assays indicated that the binding of KIF3C to MTs was elevated in H520^VASH2^ cells compared to H520^VASH2-mut^ and control cells (Fig. 5F). In addition, co-immunoprecipitation assay demonstrated that EGFR was able to interact with KIF3C after EGF stimulation, and the mount of KIF3C binding with EGFR was higher in H520^VASH2^ cells than that in H520^VASH2-mut^ cells (Fig. 5G). Collectively, these results demonstrated that KIF3C interacted with EGFR and the VASH2-induced increase in tubulin detyrosination promoted the binding of KIF3C to MTs.

It has been reported that KIF3C is considered an oncogene in several cancers [45–47]. We found KIF3C expression were higher in LUSC tissues than that in paired adjacent tissues based on TCGA database (Fig. 5H). The increased levels of KIF3C mRNA were associated with poor prognosis of LUSC patients in both TCGA cohort (Fig. 5I) and TJCH cohort (Fig. 5J). Moreover, the protein levels of KIF3C negatively correlated with patient prognosis (Fig. 5K) and positively correlated with VASH2 in SupCh cohort, detected by IHC assays (Fig. 5L). Our data suggested that KIF3C could play an important role in LUSC progression.

### KIF3C knockdown attenuated the VASH2-enhanced EGFR-endosomal recycling as well as LUSC progression and chemoresistance

**Fig. 6:**
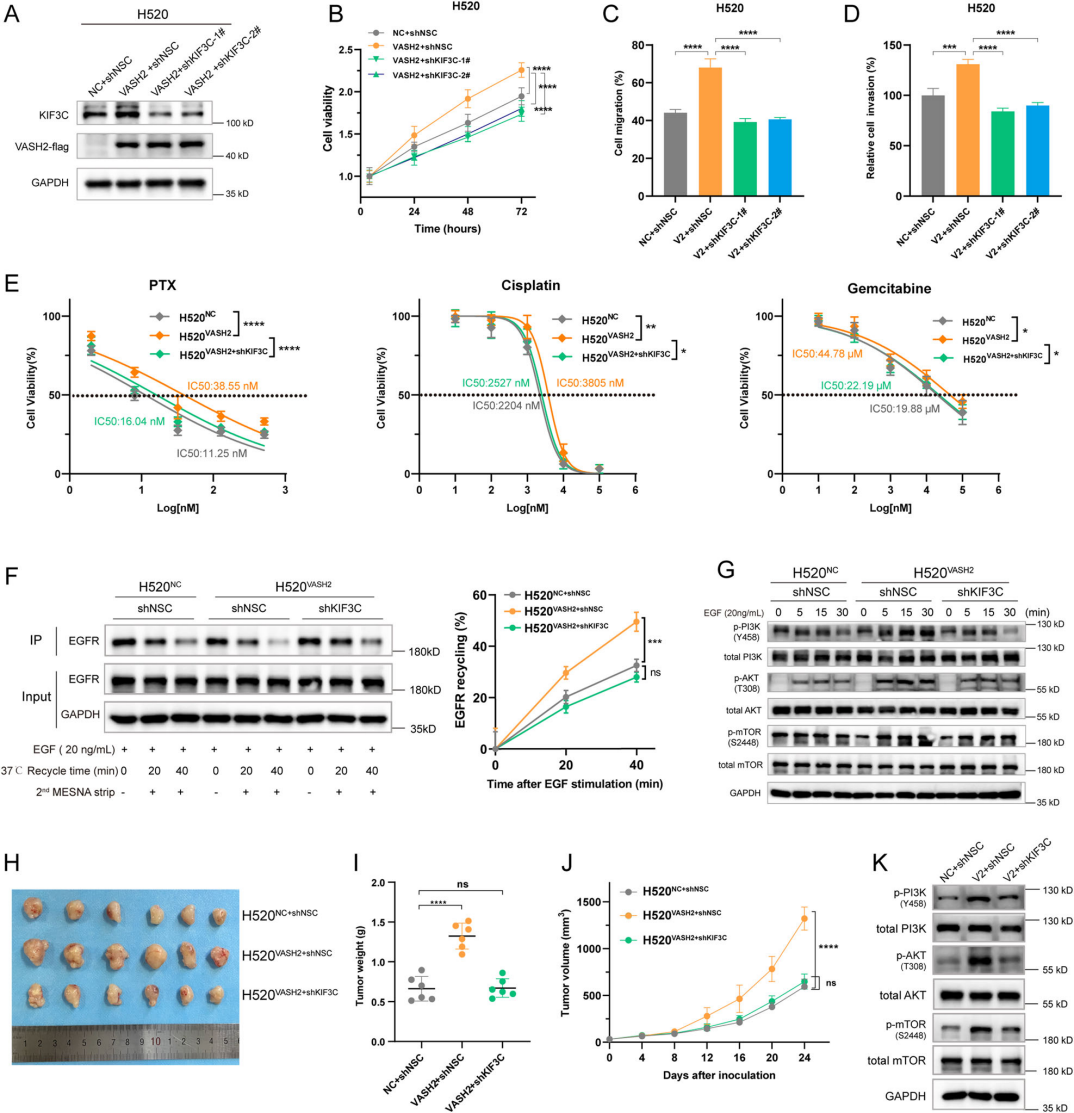
KIF3C knockdown rescued the enhancement of EGFR recycling and LUSC cell proliferation and invasion caused by VASH2. **A** KIF3C protein levels were detected after transfection of shKIF3C-1^#^ or shKIF3C-2^#^ in H520^VASH2^ cells. **B**–**D** KIF3C knockdown attenuated VASH2-caused increase in cell proliferation (**B**), cell migration (**C**) and cell invasion (**D**). **E** The IC_50_ values of PTX, cisplatin and gemcitabine were decreased in H520^VASH2+shKIF3C^ cells compared to H520^VASH2^ cells. **F** Representative images of western blotting (left) and quantification (right) of EGFR recycling in the indicated cells. **G** Representative images of western blotting for the phosphorylation of PI3K/Akt/mTOR pathway in the indicated cells. **H**–**J** General view (**H**), tumor weight (**I**) and tumor volume (**J**) of xenograft tumors formed by H520^NC+shNSC^, H520^VASH2+shNSC^ and H520^VASH2+shKIF3C^ cells (six mice per group). (**K**) Representative images of western blotting for the phosphorylation of PI3K/Akt/mTOR in the indicated xenograft tumors. Data were representative of three independent experiments. Groups compared using one-way ANOVA analysis, *, *p* < 0.05; **, *p* < 0.01; ***, *p* < 0.001 and ****, *p* < 0.0001.

To determine the role of KIF3C in VASH2-related LUSC tumorigenesis, we performed a rescue experiment by knocking down KIF3C in H520^VASH2^ cells. The protein levels of KIF3C were reduced by transfection with the shKIF3C-1^#^ or shKIF3C-2^#^ vector in H520^VASH2^ cells (Fig. 6A). The increased cell proliferation (Fig. 6B), migration (Fig. 6C) and invasion (Fig. 6D) of H520^VASH2^ cells were recovered by KIF3C silence. And knockdown of KIF3C decreased the VASH2-induced chemoresistance to paclitaxel, cisplatin and gemcitabine in H520 cells (Fig. 6E). Importantly, results showed that the enhanced EGFR-recycling rate caused by VASH2 overexpression was rescued by silencing KIF3C (Fig. 6F), and the prolonged activation of PI3K/Akt/mTOR pathway in H520^VASH2^ cells was decreased after KIF3C knockdown (Fig. 6G).

Similar results were obtained from xenograft tumor models in vivo (Fig. 6H, I). The increased growth of H520^VASH2^-xenograft tumors was reduced by KIF3C knockdown (Fig. 6J), and silence of KIF3C eliminated the elevation of PI3K/Akt/mTOR phosphorylation in H520^VASH2^-xenograft tumors (Fig. 6K). Taken together, these findings illustrated that KIF3C knockdown blocked the VASH2-induced enhancement of EGFR endosomal recycling and rescued LUSC tumor progression and chemoresistance.

## Discussion

VASH2 has been considered an angiogenic factor which promotes tumor progression by accelerating tumor angiogenesis in various cancers [8–11], but other roles of VASH2 in tumor cells are poorly understood. In the present study, we proposed a novel hypothesis to elucidate the onco-promotive effect of VASH2 besides inducing angiogenesis and lymphangiogenesis which implied that VASH2 might be a extremely important coordinator to manipulate the EGFR endosomal recycling in LUSC cells by regulating the detyrosination of microtubules.

Studies have reported that EGFR overexpression is observed in NSCLC, more frequently in LUSC than in LUAD (82% vs 44%), but its prognostic value remains controversial [4, 48]. Though EGFR did not act as a prognostic indicator in LUSC patients of TCGA, TJCH and SupCh cohorts (Fig. S5A), but EGFR combined with VASH2 was capable to predict the overall survival of LUSC patients (Fig. S5B). As we all know, EGFR signaling is triggered by the binding of growth factor ligand which induces internalization of EGFR into the early endosomes, the main sorting station [37, 49]. EGFR are sorted to recycling endosomes that recycle back to the plasma membrane, contributing to EGFR signaling enhancement [36, 38, 50]. Alternatively, EGFR signaling is terminated by delivering receptors to the lysosomes for degradation, a process known as downregulation [51, 52]. The fate of EGFR signaling is mainly determined by the balance between the degradation of endocytotic EGFR in lysosomes and EGFR recycled back to the cell surface [53, 54]. We found that the VASH2-induced increase in detyrosination of α-tubulin promoted EGFR endosomal recycling but had little impact on early-to-late EGFR endosomal maturation and degradation, which facilitated the more efficient reuse of EGFR in LUSC cells.

Endocytic EGFR in endosomes, before delivery to intraluminal vesicles and lysosomes, may remain engaged with ligand and its cytoplasmic domain may continue signaling, which is generalized as signaling endosomes [36, 55]. Signaling endosomes, serving as a platform for sustained receptor signaling and specific signaling inside the cell, not only continue the signaling processes initiated at the cell surface but also gain access to new signaling molecules and start new signaling processes [56, 57]. It has been previously shown that faster EGFR recycling to the membrane enables the cancer cell to sustain PI3K/Akt activation and cancer progression [38, 58, 59], which is in agreement with our findings in LUSC cells.

Accumulating evidence illustrates that alterations in PTMs of MTs cause global effects on intracellular signal transduction and cancer pathogenesis [17, 18, 60, 61]. Wasylyk et al. showed that altered tubulin polyglutamylation was linked to tumorigenesis and resistance to chemotherapeutic drugs in prostate cancer [62, 63]. Other studies reported that EGFR trafficking and degradation was regulated by HDAC6 and tubulin acetylation [64, 65]. Tubulin acetylation modulates the MT-based transport of Hsp90-chaperoned proteins and Akt kinase signaling [66], and using HDAC inhibitor to enhance the level of tubulin acetylation could reduce PI3K/Akt/mTOR signaling [67]. Whereas, little is known about the role of tubulin detyrosination in EGFR endosomal trafficking or downstream signal transduction in cancers. The present study first revealed that VASH2-induced increase in tubulin detyrosination facilitated EGFR recycling and prolonged the activation of downstream PI3K/Akt/mTOR signaling, leading to promote the malignant biological behaviors of LUSC cells and chemoresistance. This may be the underlying mechanism that why increased levels of tubulin detyrosination were negatively correlated with LUSC patient prognosis (Fig. 2), which was consistent with previously reported studies that abnormal levels of tubulin detyrosination were associated with tumor progression [19, 20].

Vasohibins, including VASH1 and VASH2, have been initially identified as angiogenic factors. VASH1 and VASH2 have been reported to function differently, with VASH1 inhibiting angiogenesis and tumor metastasis, whereas VASH2 promotes angiogenesis and tumor progression [60, 61]. However, the contradictory roles of VASH1 and VASH2 in cancers are remaining largely unknown, particularly in association with microtubule detyrosination. The study by Kobayashi et al. showed that VASH1-induced tubulin detyrosination inhibited angiogenesis by impairing the endocytosis and trafficking of VEGF-receptor 2 (VEGFR2) in endothelial cells [68], which was contrary to our findings that VASH2-induced tubulin detyrosination promoted LUSC tumor growth by facilitating the EGFR recycling in cancer cells. This suggests that VASH1- and VASH2-induced microtubule detyrosination has different modes of action and is cell type-dependent.

The overall homology between VASH1 and VASH2 is only 52.5% at the amino acid level [69], which contributes to their difference in a three-dimensional structure and the molecular interaction. Recent study by Ramirez-Rios et al. have demonstrated that the divergent mode of detyrosination between VASH1 and VASH2 is correlated with the microtubule-binding properties of their disordered N-terminal and C-terminal regions. VASH2 have a different microtubule-binding configuration of its central catalytic region compared to VASH1, thus VASH1 and VASH2 could generate distinct microtubule subpopulations and confined areas of detyrosinated lattices [70]. Moreover, the different microtubule subpopulations can attract distinctive subsets of microtubule-associated proteins, which regulate their interactions in a specific and selective manner and drive various microtubule-based cellular functions [71].

It is well known that endosomal vesicles containing the EGF-EGFR complex are delivered to their intermediate or final destinations through interaction with motor proteins along MT tracks, which include cytoplasmic dynein and kinesin proteins [44, 64]. The dynein-dynactin complex is mainly responsible for retrograde transport from cell periphery to perinuclear, while most kinesin family members direct the anterograde transport of cargoes to the cell surface [40, 41]. Studies have reported that the kinesin-2 complex was involved in the long-range transport of low-density lipoprotein receptor recycling endosomes to plasma membrane along MT tracks [39], and regulated endocytic receptor recycling through its interaction with RAB11-Family Interacting Protein 5 [42]. In our study, we found KIF3C, a member of the kinesin-2 family, interacted with EGFR, and the VASH2-induced increase in detyrosination of α-tubulin boosted the recruitment of KIF3C to MTs in LUSC cells (Fig. 5).

Although KIF3C is reported to promote tumor development in several cancers [45–47], the molecular mechanism remains elusive. Our results demonstrated that the VASH2-enhanced EGFR recycling and PI3K/Akt/mTOR activation were rescued by KIF3C knockdown (Fig. 6). The silence of KIF3C attenuated VASH2-caused chemoresistance in LUSC cells and decreased H520^VASH2^-xenograft tumor growth (Fig. 6). This study revealed the mechanism of KIF3C in LUSC tumorigenesis by showing that the VASH2-induced increase in tubulin detyrosination enhanced the KIF3C-dependent endosomal recycling of EGFR and sustained the activation of downstream PI3K/Akt/mTOR signaling.

It has been demonstrated that “tubulin code” is able to affect the affinity, velocity and processivity of MT motors [43, 72–78]. Vesicle trafficking along MT tracks not only depends on the motor-cargo complex but can also be modulated through PTM of tubulin. Tubulin polyglutamylation differentially regulates the MT-interacting proteins kinesin-1, Tau and katanin [79]. Both tyrosinated and detyrosinated MTs can attract specific subsets of microtubule-associated proteins; for instance, mitotic centromere-associated kinesin is attracted to tyrosinated MTs [77], whereas the kinesin motors centromere-associated protein E and kinesin-2 preferentially bind to detyrosinated MTs [43, 76]. Removal of the C-terminal tyrosine of α-tubulin increases the velocity and processivity of kinesin-2 by ~2 and 2.5-fold, respectively, which suggest that it is available to regulate kinesin-2-mediated vesicle transport by preferentially binding with detyrosinated MTs [43]. This is in accordance with our findings that VASH2-induced tubulin detyrosination boosted the binding of KIF3C to MTs in LUSC cells, and that deY-tubulin-rich MTs are suitable as traffic rails for anterograde transport of EGFR endosomes.

Chemotherapy is still the main treatment for LUSC patients. However, several studies reported that VASH2 decreased chemosensitivity in cancers [80–82], which are in agreement with our findings in LUSC cells, including paclitaxel, cisplatin and gemcitabine (Figs. 3 and S2). This might be caused by VASH2-enhanced activation of PI3K/Akt/mTOR signaling which is often associated with multi-drug resistance [48–52]. PI3K/Akt/mTOR pathway renders a survival signal to withstand cytotoxic anticancer drugs and enhances cancer stem cell characteristics. By using the xenograft tumor mouse model, we found that PI3K/Akt/mTOR activation in tumor was obviously reduced by using EpoY. Moreover, paclitaxel treatment efficacy on LUSC was remarkably improved in combination with EpoY in vivo (Fig. 3), which suggested that blocking TCP activity of VASH2 could inhibit LUSC tumor growth and increase the sensitivity of chemotherapeutic drugs. In addition, EpoY decreased xenograft tumor growth by suppressing LUSC cell proliferation and tumor angiogenesis, indicated by the levels of proliferative Ki67+ cells and the microvessel density of CD31 (Fig. S2). VASH2 was identified as a secreted protein, and our findings showed that intracellular overexpression of VASH2 protein promoted the secretion of soluble VASH2 protein into the supernatants. Importantly, EpoY reduced VASH2 secretion into the supernatants in a dose-dependent manner (Fig. S7), suggesting that TCP inhibitor may inhibit the pro-angiogenic effects of VASH2 in the tumor microenvironment by reducing the secretion of soluble VASH2 in LUSC cells. Altogether, these results imply that, besides serving as an angiogenic factor, VASH2 also promotes LUSC cell proliferation and chemoresistance, which suggests that VASH2 could play a dual role in LUSC treatment.

LUAD is another common type of non-small cell lung cancer, our results showed that VASH2 expression was not significantly elevated in LUAD tissues compared to adjacent normal tissues (Fig. S8A) and had no significant correlation with the overall survival prognosis of LUAD patients (Fig. S8B). However, the overexpression of VASH2 significantly promoted cell proliferation, migration, and invasion, while inhibiting cell apoptosis in A549 cells. Notably, the pro-oncogenic effects of VASH2 in A549 cells were significantly attenuated when its enzymatic activity was disrupted by mutation (Fig. S8C–G). These findings indicate that although overexpression of VASH2 can promote malignant biological behaviors of A549 cells by enhancing microtubule detyrosination, the fact that VASH2 is not significantly increased in LUAD tissues suggests that its role in LUAD is not dominant. However, further in-depth researches are needed to explore the role of TCP activity of VASH2 in LUAD in the future.

**Fig. 7:**
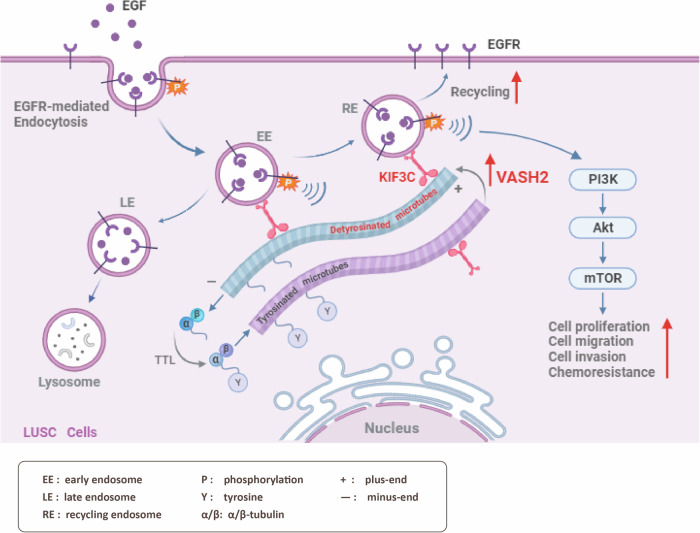
A proposed model depicts how VASH2 promotes chemoresistance and the malignant biological behaviors of LUSC cells by increasing the detyrosination of α-tubulin. VASH2-induced increase in tubulin detyrosination boosts the binding of KIF3C to microtubules and enhances KIF3C-dependent endosomal recycling of EGFR, contributing to the prolonged activation of downstream PI3K/Akt/mTOR signaling and LUSC progression.

In summary, our findings unveil, for the first time to the best of our knowledge, that VASH2 enhanced KIF3C-mediated endosomal recycling of EGFR by increasing the detyrosination of α-tubulin and sustained the activation of PI3K/Akt/mTOR signaling, contributing to LUSC progression and chemoresistance (Fig. 7). Our findings demonstrated that VASH2, besides serving as an angiogenic factor, was able to promote malignant biological behaviors and chemoresistance of LUSC cells by its tubulin carboxypeptidase activity. This study suggests that VASH2 is not only a prognostic biomarker but also a promising therapeutic target for LUSC patients, which offers a novel insight that a combination of chemotherapy and the TCP inhibitor may be a promising treatment strategy for LUSC patients.

## Supplementary information

Supplementary Figures and Tables

Supplementary original WB data

## Data Availability

The datasets used and/or analyzed during the current study are available from the corresponding author upon reasonable request.
