# Supplementary material for: VASH2 enhances KIF3C-mediated EGFR-endosomal recycling to promote aggression and chemoresistance of lung squamous cell carcinoma by increasing tubulin detyrosination
Source: Cell Death Dis. 2024 Oct 23;15(10):772. doi: 10.1038/s41419-024-07155-x (PMC11499603; doi:10.1038/s41419-024-07155-x)
Supplement: Supplementary file 1 — Supplementary Figures and Tables [file 41419_2024_7155_MOESM1_ESM.pdf]

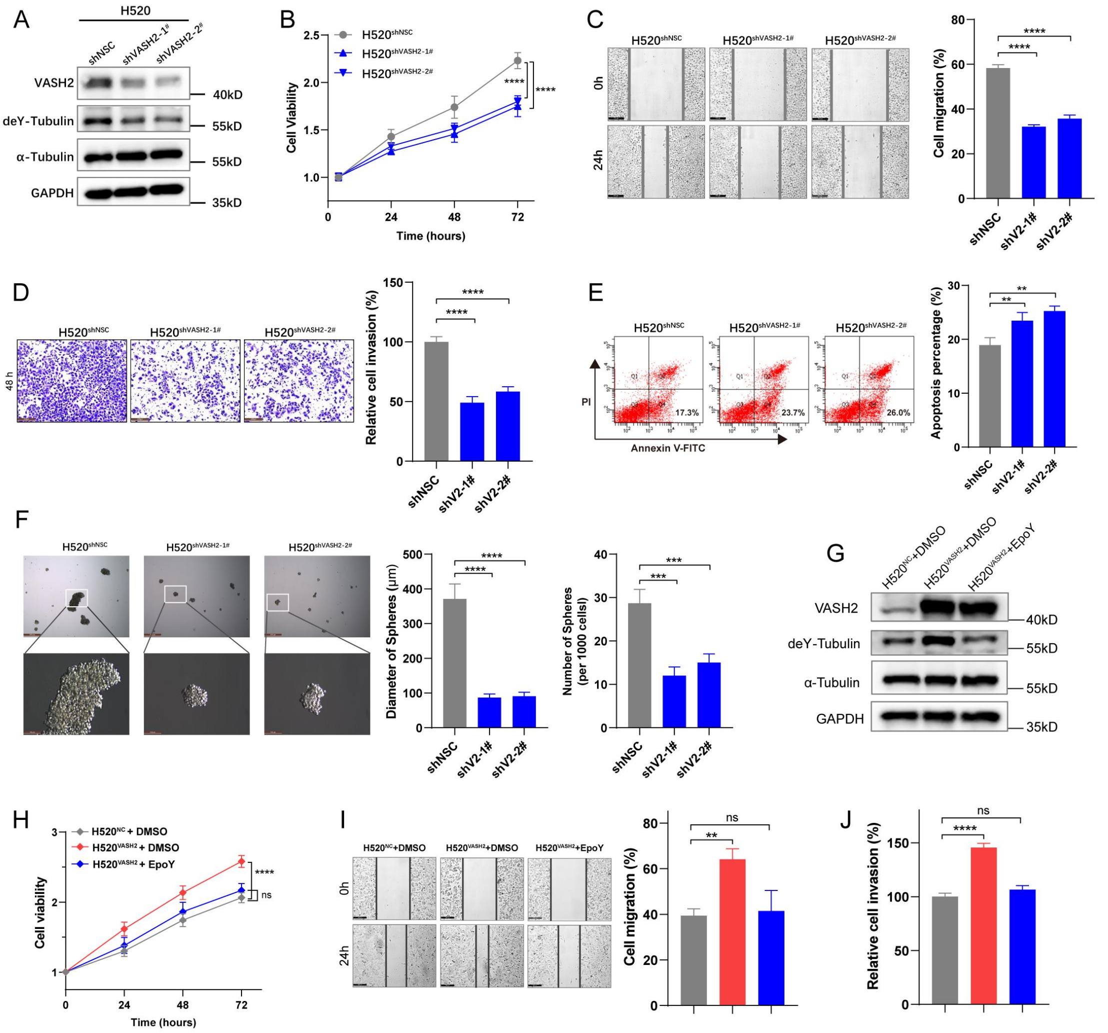

Fig. S1 VASH2 regulated the malignant biological behaviors of H520 cells by inducing detyrosination of α-tubulin. (A) Western blotting showed deY-tubulin was decreased by shVASH2 in H520 cells. (B) Cell proliferation of H520 was suppressed by shVASH2. (C) Representative images (left) and cell migration quantification (right) of wound healing assays after VASH2 knockdown in H520 cells. Scale bar, 200 μm. (D) Representative images (left) and cell invasion quantification (right) of trans-well assays after VASH2 knockdown in H520 cells. Scale bar, 200 μm. (E) H520 cell apoptosis was increased by shVASH2, determined by Annexin V-FITC assays. (F) Representative images of H520 sphere formation assays after VASH2 knockdown (left) and quantification based on diameter and number of spheres (right). (G) The increased deY-tubulin caused by VASH2 was reduced by using inhibitor EpoY (10 μM). (H) VASH2-related cell proliferation was suppressed by adding inhibitor EpoY (10 μM). (I) Representative images (left) and cell migration quantification (right) of wound healing assays after adding inhibitor EpoY (10 μM) in $H520^{VASH2}$ cells. Scale bar, 200 μm. (J) Cell invasion ability was decreased by using inhibitor EpoY (10 μM) in $H520^{VASH2}$ cells. Data were representative of three independent experiments.The One-Way ANOVA tests, *, $p < 0.05$; **, $p < 0.01$; ***, $p < 0.001$ and ****, $p < 0.0001$.

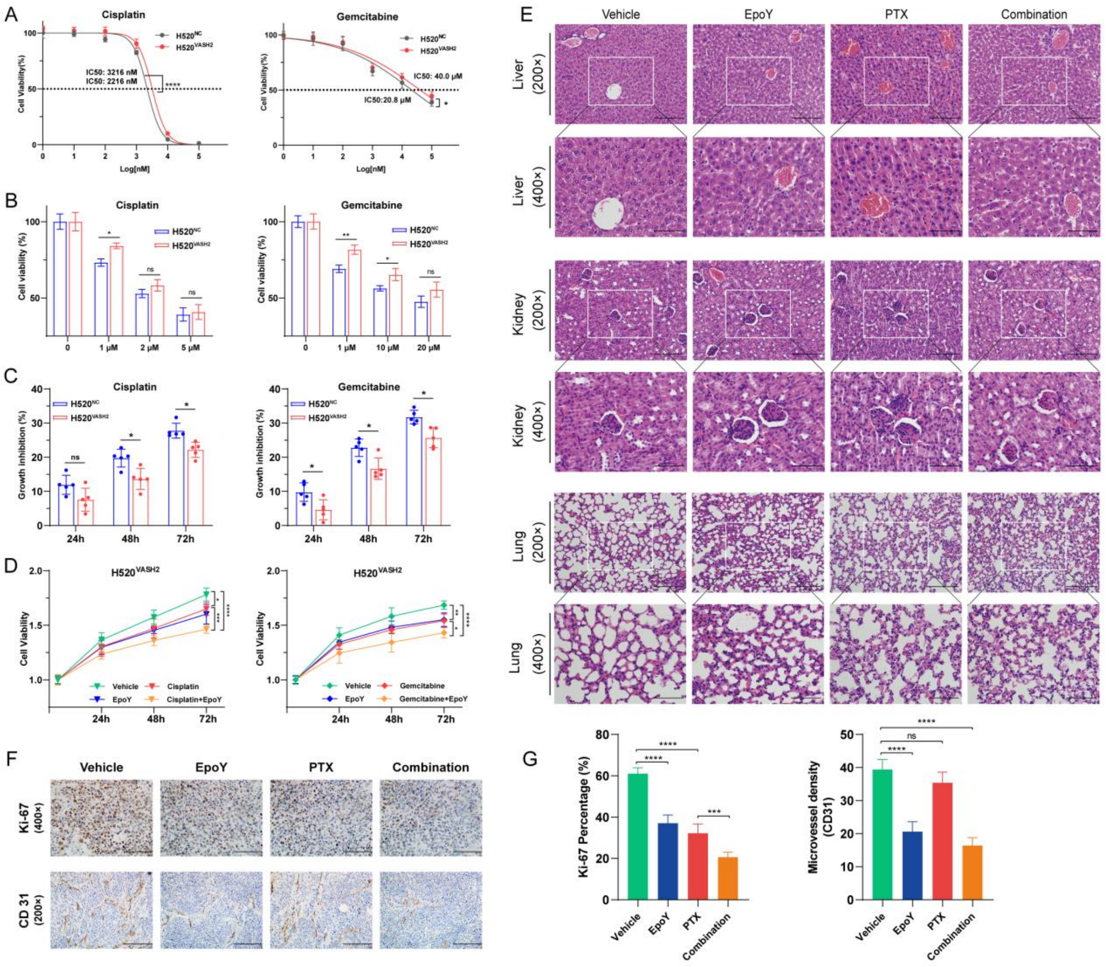

Fig. S2 EpoY reduced the VASH2-induced chemoresistance in H520 cells. (A) The IC_{50} values of cisplatin (left) and gemcitabine (right) in $H520^{NC}$ and $H520^{VASH2}$ cells were determined 48 h post-treatment. (B) Cell viability was measured by treatment with different concentrations of cisplatin (left) or gemcitabine (right) in $H520^{NC}$ and $H520^{VASH2}$ cells for 48 h. (C) $H520^{NC}$ and $H520^{VASH2}$ cells were treated with 1 μM cisplatin (left) or 1 μM gemcitabine (right) for 24 h, 48 h and 72 h. Cell viability was evaluated by CCK-8 assay. (D) Anticancer activity of cisplatin (1 μM), gemcitabine (1 μM), and gemcitabine-EpoY combination against $H520^{VASH2}$ cells. (E) Representative images of hematoxylin-eosin staining of mice liver, kidney and lung. Scale bar, 50 μm (upper), 100 μm (lower) (F) Representative images of immunohistochemistry staining of Ki-67 and CD31 in $H520^{VASH2}$-xenograft tumors with PTX and/or EpoY treatment. Scale bar, 100 μm (upper), 50 μm (lower). (G) Quantification of Ki-67 percentage and MVD of CD31. The One-Way ANOVA tests, *, $p < 0.05$; **, $p < 0.01$; ***, $p < 0.001$ and ****, $p < 0.0001$.

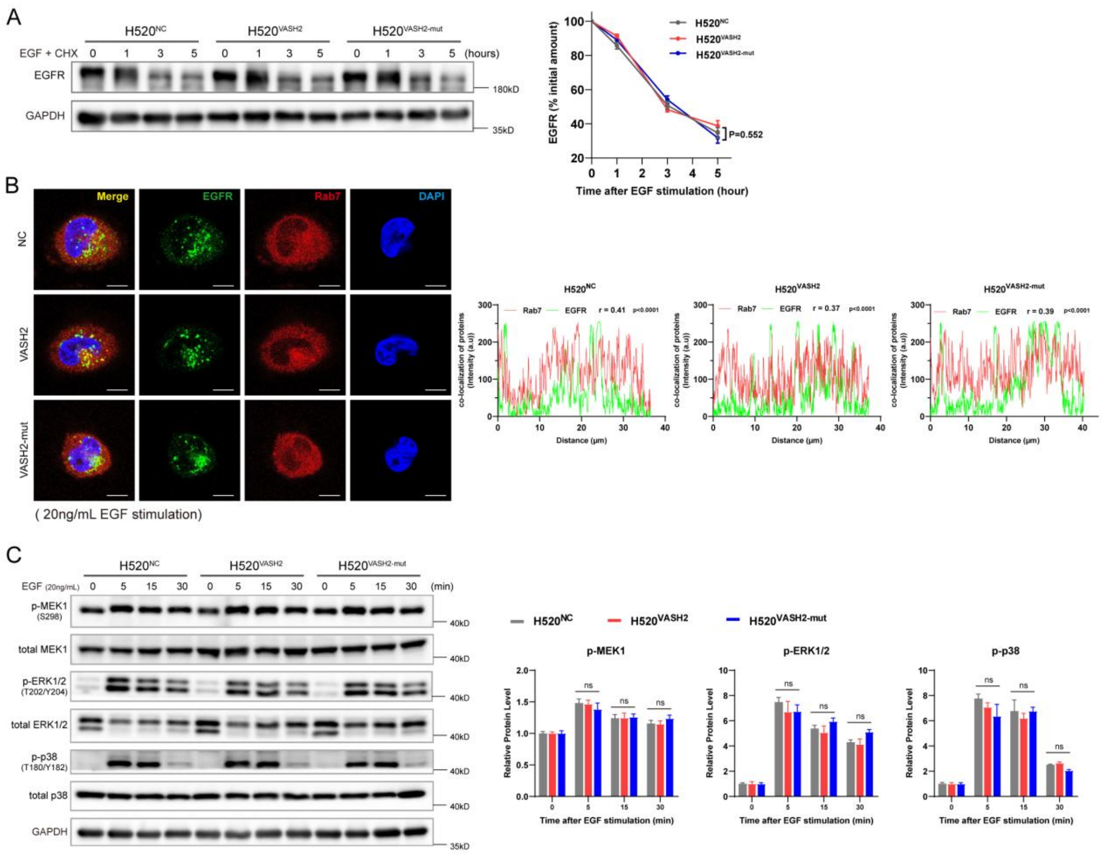

Fig. S3 VASH2-induced MT detyrosination had little impact on EGFR degradation and MAPK pathway activation. (A) EGFR degradation assays with cycloheximide (50 µg/mL) and EGF (20 ng/mL) were conducted at 37°C for 0 h, 1 h, 3 h and 5 h. Representative images of western blotting (left) and quantification (right) of the remaining EGFR. (B) Representative images of immunofluorescence assays (left) and the co-localization analysis (right) of EGFR and Rab7 in $H520^{NC}$, $H520^{VASH2}$ and $H520^{VASH2\text{-}mut}$ cells. Scale bar, 10 µm. (C) Western blotting (left) and quantification (right) for the phosphorylation of MEK1,ERK1/2 and p38 proteins in the indicated cells. Data were representative of three independent experiments. Groups compared using one-way ANOVA analysis, ns, $p > 0.05$.

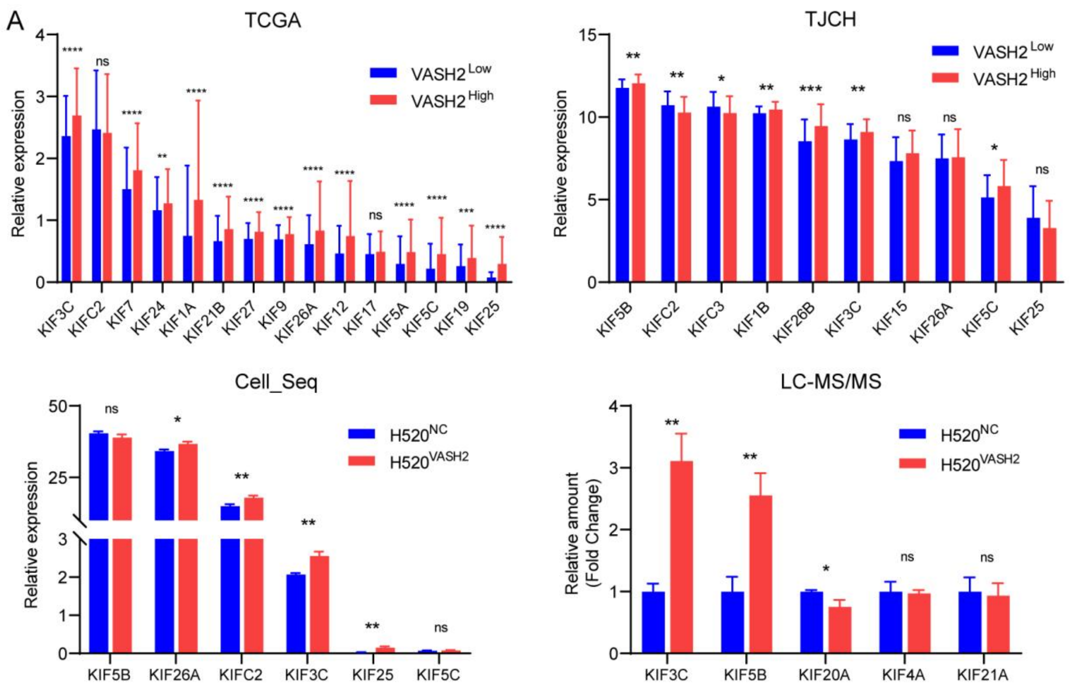

Fig. S4 KIF3C was highly expressed in VASH2^{high} LUSC cells and tissues. (A) Screening for the VASH2-related differentially expressed KIFs from LUSC tissues and cells, including RNA-seq analysis of the LUSC tissues from the TCGA cohort and TJCH cohort, as well as LC-MS/MS (α-tubulin-immunoprecipitated samples) and RNA-seq analysis of H520^{VASH2} and H520^{NC} cells. The student's t tests between two groups, *, $p < 0.05$; **, $p < 0.01$; ***, $p < 0.001$ and ****, $p < 0.0001$.

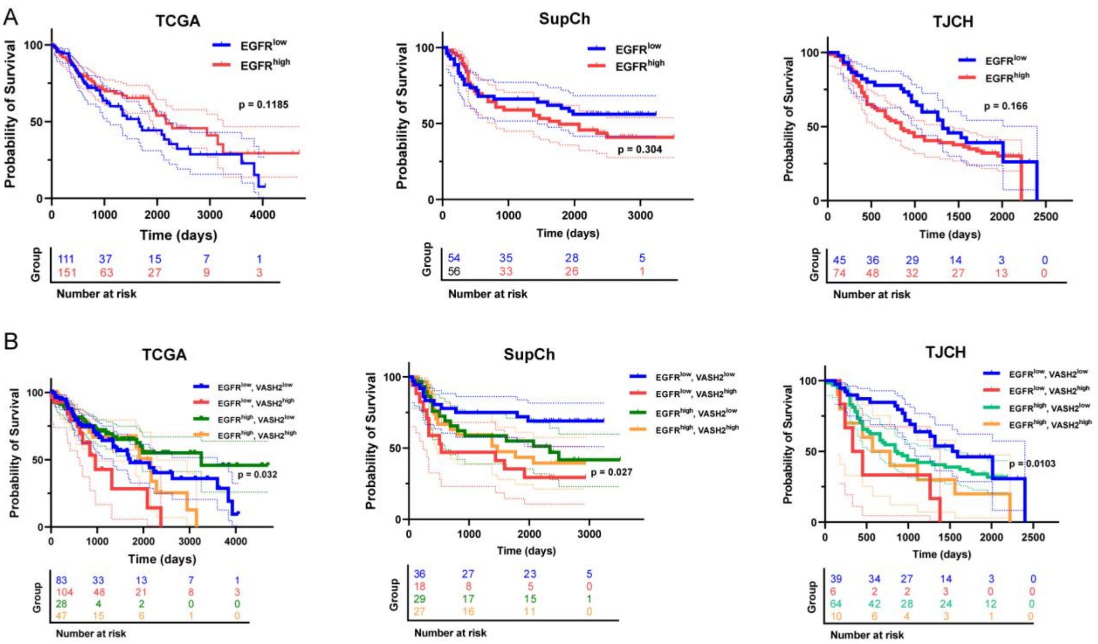

Fig. S5 Overall survival of LUSC patients stratified based on EGFR and VASH2 expression. (A) Kaplan-Meier curves representing the overall survival of LUSC patients stratified based on EGFR expression levels in TCGA cohort, SupCh cohort and TJCH cohort. (B) Kaplan-Meier curves representing the overall survival of LUSC patients stratified based on EGFR and VASH2 expression levels in TCGA cohort, SupCh cohort and TJCH cohort. p values were calculated by log-rank test.

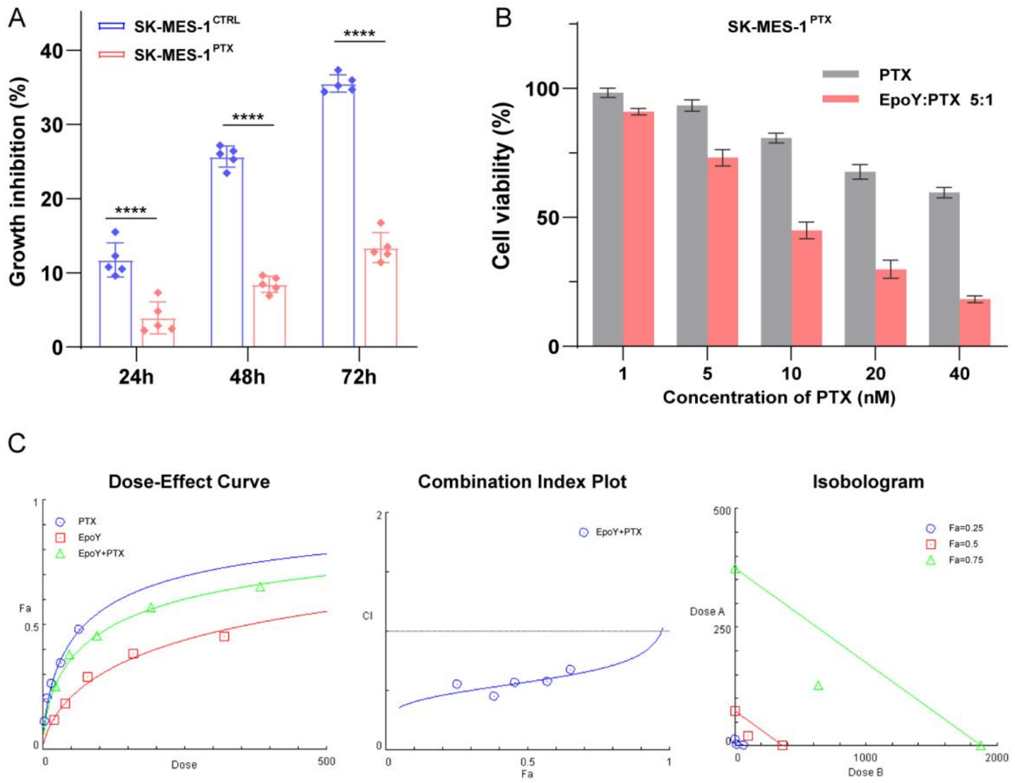

Fig. S6 Synergistic effect of paclitaxel and EpoY combination against SK-MES-1^{PTX} cells. (A) CCK-8 assays were performed at 24 h, 48 h and 72 h post-treatment with 10 nM paclitaxel in SK-MES-1^{CTRL} and SK-MES-1^{PTX} cells. (B) Cell viability in SK-MES-1^{PTX} was determined at 48 h post-treatment with paclitaxel alone and EpoY-paclitaxel combination. Cells treated with different concentrations of paclitaxel, and the combination with EpoY using the mass ratios of 1:5. (C) Dose-effect curve of paclitaxel and EpoY, Combination index (CI) plot of paclitaxel-EpoY combination, isobologram of paclitaxel-EpoY combination at Fa=0.25, 0.5 and 0.75, obtained from the CompuSyn analysis. Fa, fractional inhibition.

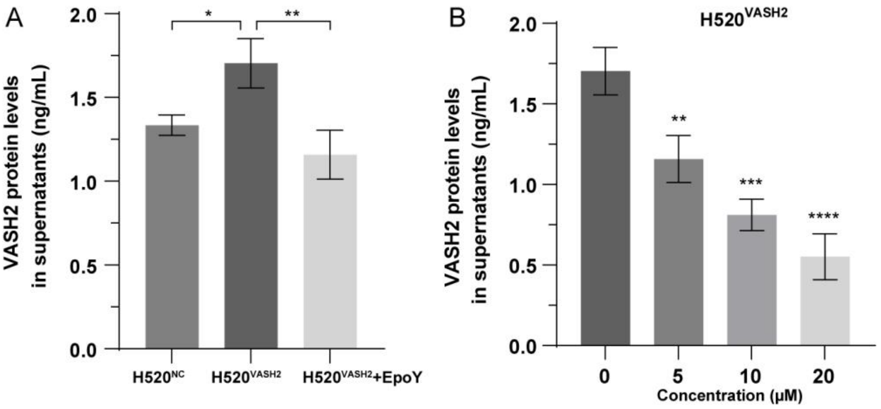

Fig. S7 EpoY inhibited the secretion of soluble VASH2 into the supernatants. (A) The protein levels of VASH2 in cell culture supernatants of H520^{NC}, H520^{VASH2} and H520^{VASH2} cells treated with 5 μM EpoY for 24 h. (B) Determination of VASH2 protein levels in the supernatants from H520^{VASH2} cells treated with EpoY at concentrations of 0, 5, 10, 20 μM for 24 h.

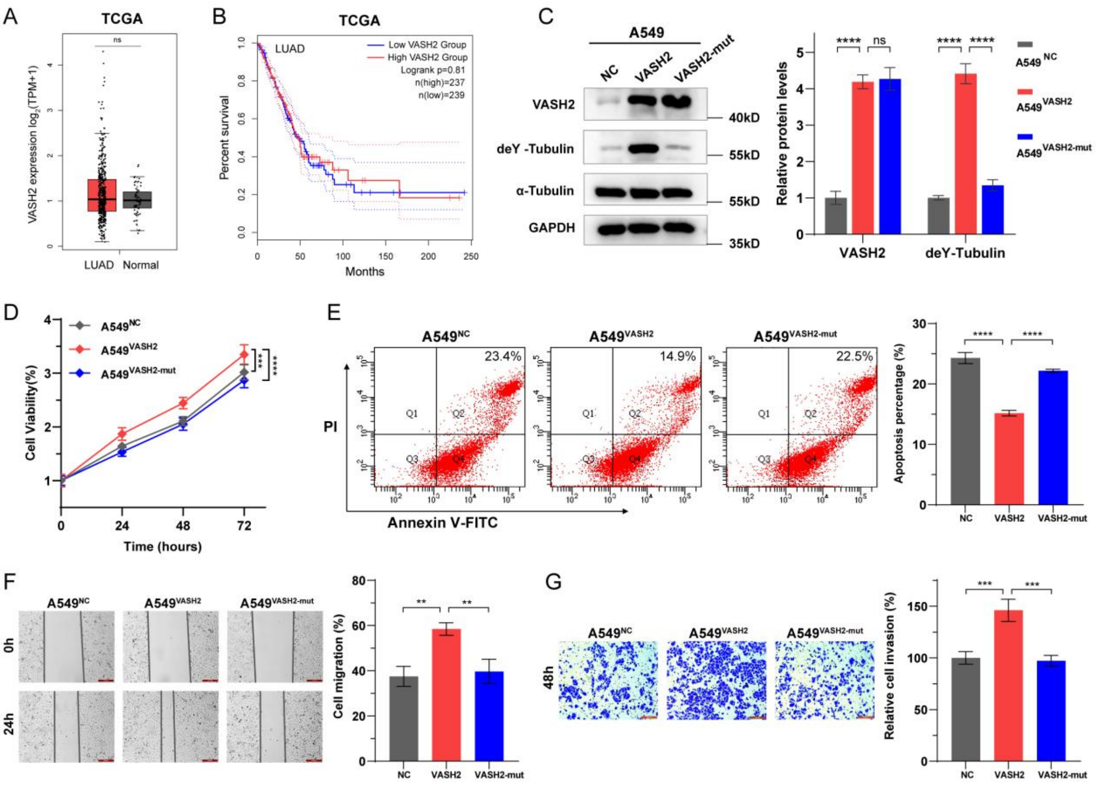

Fig. S8 VASH2 promoted the malignant biological behaviors of A549 cells through TCP activity. (A) The expression levels of VASH2 in LUAD tissues and the para-carcinoma tissues of TCGA datasets analyzed by GEPIA2. (B) Overall survival of LUAD patients stratified based on VASH2 expression in TCGA datasets analyzed by GEPIA2. (C) Representative images of western blotting and quantification for the expression of VASH2 and deY-tubulin after overexpression of VASH2 or VASH2-C158A mutant in A549 cells. GAPDH was used as an internal control. (D) Cell proliferation of A549^{VASH2} was increased compared to A549$^{VASH2\text{-}mut}$ and A549^{NC} cells, determined by CCK-8 assays. (E) Cell apoptosis of A549^{NC}, A549^{VASH2} and A549$^{VASH2\text{-}mut}$ cells, determined by Annexin V-FITC assays. (F) Representative images (left) and cell migration quantification (right) of wound healing assays in A549^{NC}, A549^{VASH2} and A549$^{VASH2\text{-}mut}$ cells. Scale bar, 200 μm. (G) Representative images (left) and cell invasion quantification (right) of trans-well assays in A549^{NC}, A549^{VASH2} and A549$^{VASH2\text{-}mut}$ cells. Scale bar, 200 μm. Data were representative of three independent experiments. **, $p < 0.01$; ***, $p < 0.001$ and ****, $p < 0.0001$.

Table S1 Clinical pathological information of enrolled LUSC patients

Characteristic	TCGA		TJCH		SupCh	
	Number	%	Number	%	Number	%
Enrolled patients	474	-	119	-	110	-
Gender						
Male	354	74.68	90	75.63	77	70.00
Female	120	25.32	29	24.37	33	30.00
Age (years)						
< 50	22	4.64	6	5.04	8	7.27
≧50	452	95.36	113	94.96	102	92.73
Stage of disease (TNM)						
I	231	48.73	36	30.25	41	37.27
II	154	32.49	41	34.45	35	31.82
III	82	17.3	35	29.41	25	22.73
IV	7	1.48	7	5.88	9	8.18
Tumor (of TNM)						
T1	109	23.00	21	17.65	22	20.00
T2	274	57.81	57	47.90	56	50.91
T3	69	14.56	32	26.89	24	21.82
T4	22	4.64	9	7.56	8	7.27
Regional Lymph Nodes (of TNM)						
N0	304	64.14	76	63.87	67	60.91
N1	122	25.74	17	14.29	19	17.27
N2	39	8.23	26	21.85	24	21.82
N3	5	1.05	0	0.0	0	0.0
NX	4	0.84	0	0.0	0	0.0

Table S2 List of shRNA sequences used for gene knockdown

Gene name	Accession No.	Target sequence	
VASH2	NM_024749	1#	CGCCTTCTTGGCAAAGCCTTCAATA
		2#	GGACTCTGAGTGACCTCATCTTTGA
KIF3C	NM_002254	1#	GGCCGACCUGUAUGACGAAACTT
		2#	GGGAATTCCAAGAGGAGATTT

Table S3 List of antibodies used in this study

Antibody	Applications	Vendor	Catalog Number
Anti-VASH2	WB/IHC/IF	Proteintech	Cat# 67753-1-Ig
Anti-α-tubulin	WB/IHC/IF/IP	Santa Cruz	Cat# sc-69969
Anti-detyrosinated α-tubulin	WB/IHC/IF	Sigma-Aldrich	Cat# AB3201
Anti-KIF3C	WB/IHC/IF/IP	Proteintech	Cat# 14333-1-AP
Anti-Rab7	IF	Abcam	Cat# ab126712
Anti-Rab11	IF	BD	Cat# 610657
Anti-EGFR	WB/IHC/IF/IP	Proteintech	Cat# 18986-1-AP
Anti-phospho-EGFR (Y1068)	WB	Abcam	Cat# ab40815
Anti-PI3K p85	WB	Cell Signaling Technology (CST)	Cat# 4257
Anti-pPI3K p85 (Tyr458)/p55 (Tyr199)	WB	CST	Cat# 4228
Anti-Akt	WB	CST	Cat# 4691
Anti-phospho-Akt (Ser473)	WB	CST	Cat# 4060
Anti-phospho-Akt (Thr308)	WB	CST	Cat# 13038
Anti-mTOR	WB	CST	Cat# 2983
Anti-phospho-mTOR (Ser2448)	WB	CST	Cat# 5536
Anti-MEK1/2	WB	CST	Cat# 8727
Anti-phospho-MEK1 (Ser298)	WB	CST	Cat# 98195
Anti-p44/42 MAPK (Erk1/2)	WB	CST	Cat# 4695
Anti-pMAPK (Erk1/2) (Thr202/Tyr204)	WB	CST	Cat# 4370
Anti-p38 MAPK	WB	CST	Cat# 8690
Anti-p-p38 MAPK (Thr180/Tyr182)	WB	CST	Cat# 4511
Anti-FLAG	WB	CST	Cat# 14793
Anti-GAPDH	WB	CST	Cat# sc-47724
Goat anti-Mouse IgG secondary antibody	WB	CST	Cat# ab7068
Goat anti-Rabbit IgG secondary antibody	WB	CST	Cat# ab98467
Ki-67	IHC	CST	Cat# 12202
CD31	IHC	CST	Cat# 77699

Table S4 Correlation between the Clinical pathological features and expression of VASH2 or deY-tubulin

Variables	VASH2		p value	deY-tubulin		p value
	Low (n=65)	High (n=45)		Low (n=62)	High (n=48)	
Age (years)			0.8386			0.2697
< 50	5	3		6	2	
≧50	60	42		56	46	
Gender			0.2891			0.1537
Male	43	34		40	37	
Female	22	11		22	11	
Smoking status			0.5701			0.6766
Non-smoker	16	9		15	10	
Smoker	49	36		47	38	
pT			0.0196			0.1135
T1	15	7		15	7	
T2	37	19		34	22	
T3	12	12		11	13	
T4	1	7		2	6	
pN			0.0166			0.010
N0	36	31		39	28	
N1	16	2		15	4	
N2	12	12		8	16	
pTNM stage			0.1236			0.0477
I	25	16		27	14	
II	23	12		22	13	
III	15	10		11	14	
IV	2	7		2	7	

Chi-square test. $p<0.05$ was considered statistically significant. pathological T category (pT), pathological N category (pN), pathological stage (pStage).
