# Supplementary figures and images for: VASH2 enhances KIF3C-mediated EGFR-endosomal recycling to promote aggression and chemoresistance of lung squamous cell carcinoma by increasing tubulin detyrosination
Source: Cell Death Dis. 2024 Oct 23;15(10):772. doi: 10.1038/s41419-024-07155-x (PMC11499603; doi:10.1038/s41419-024-07155-x)

Fig. 1E

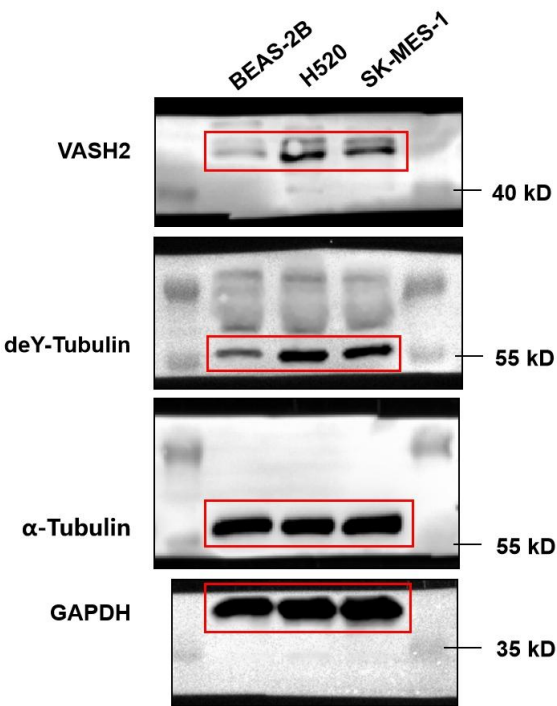

Fig. 1F

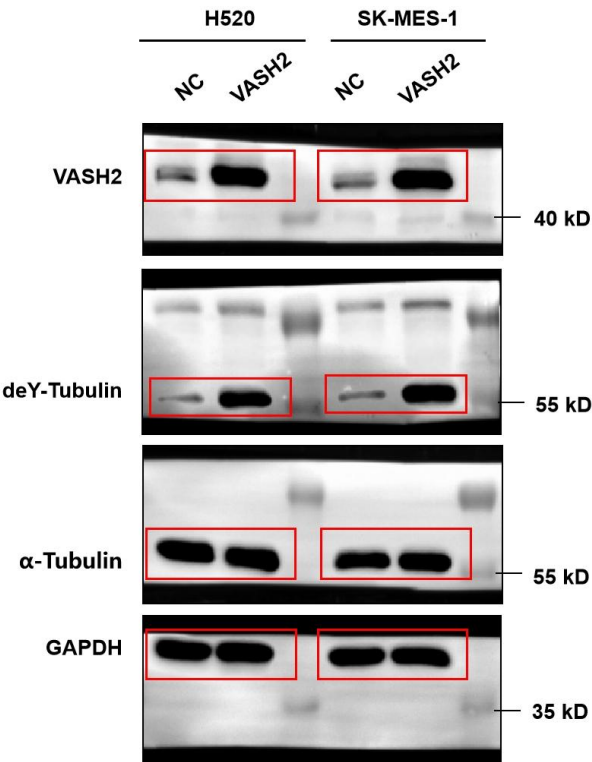

Fig. 2E

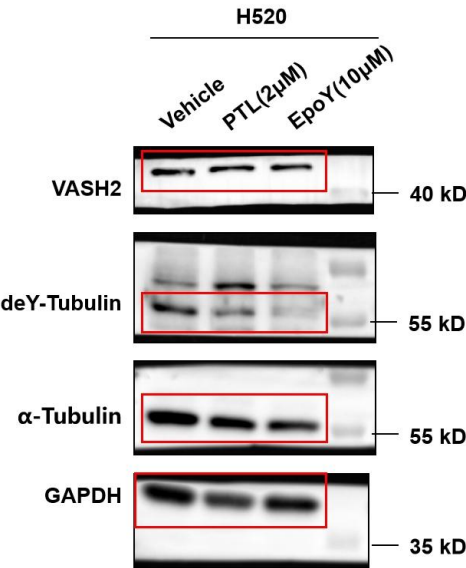

Fig. 3P

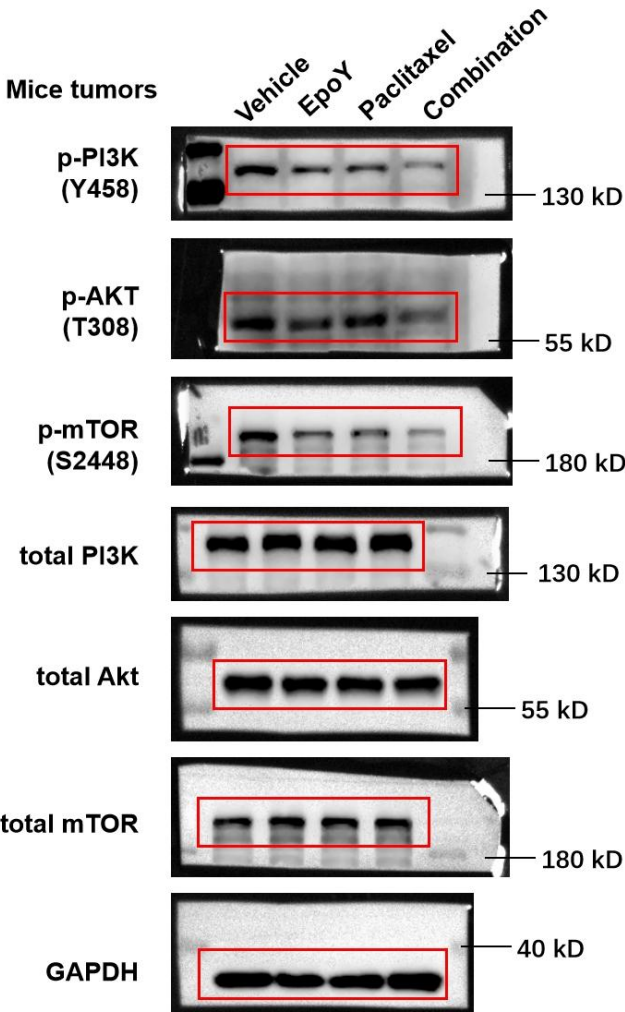

Fig. 4A

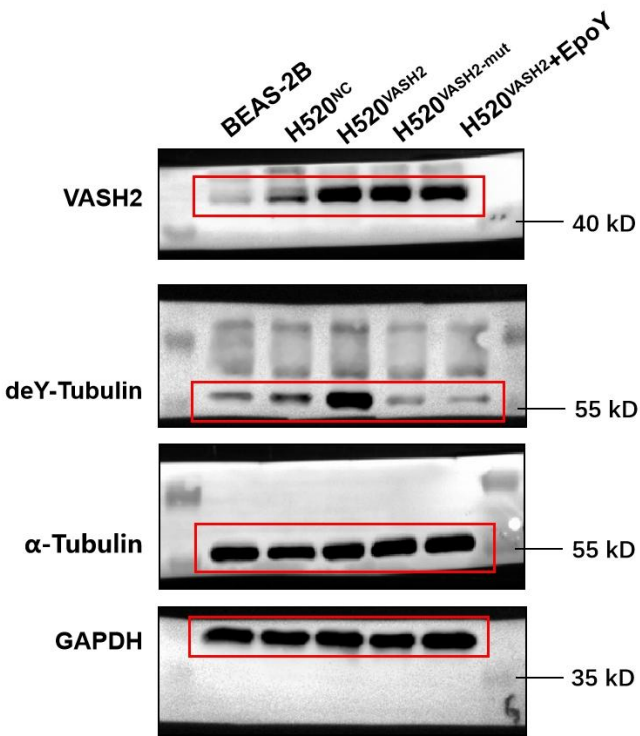

Fig. 4G

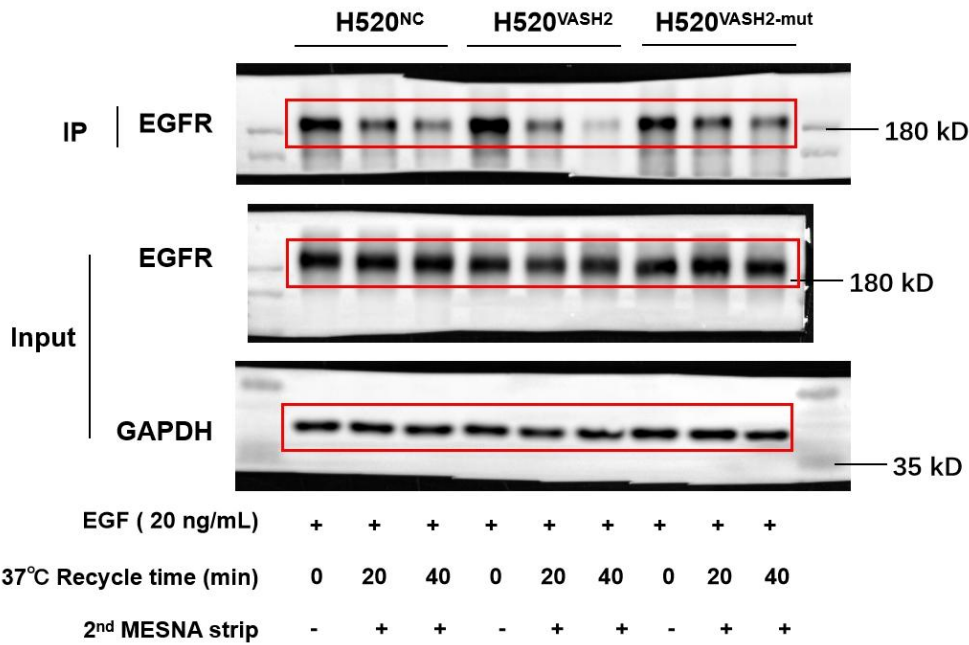

**Fig. 4J**

Fig. 5F

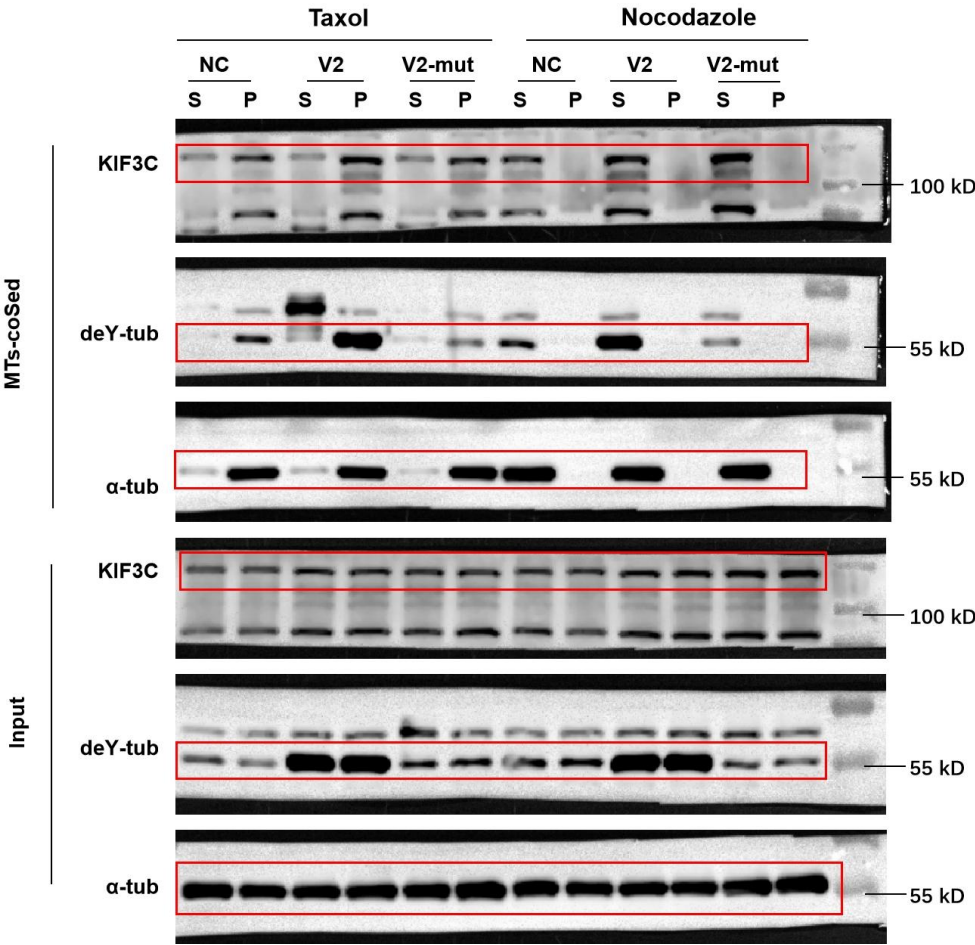

Fig. 5G

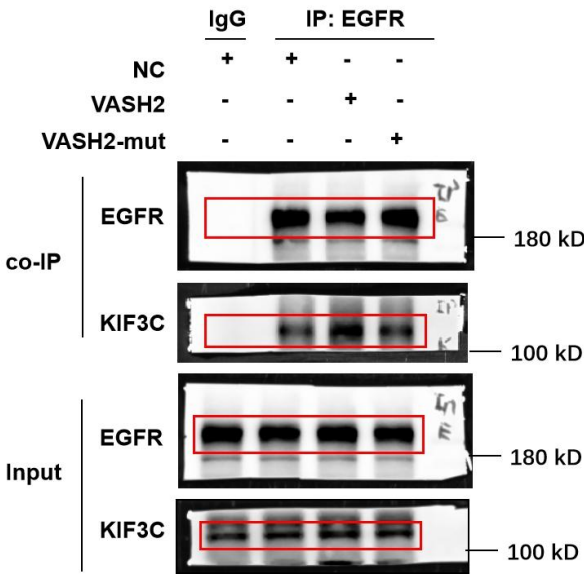

Fig. 6A

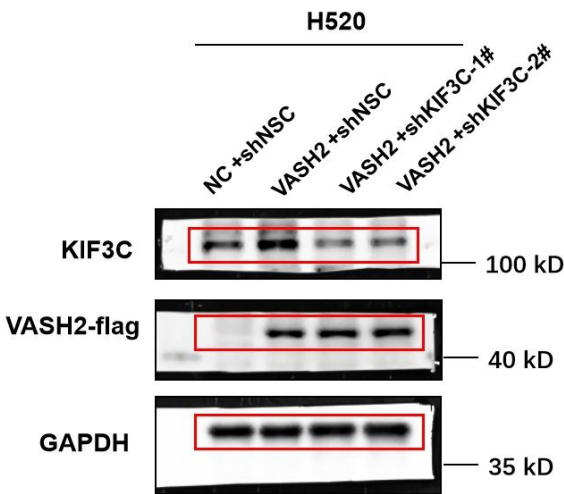

Fig. 6F

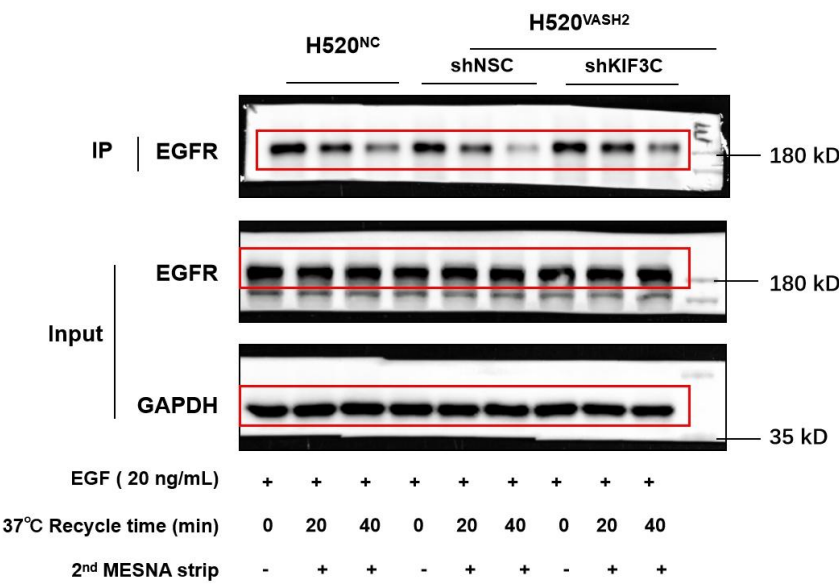

Fig. 6G

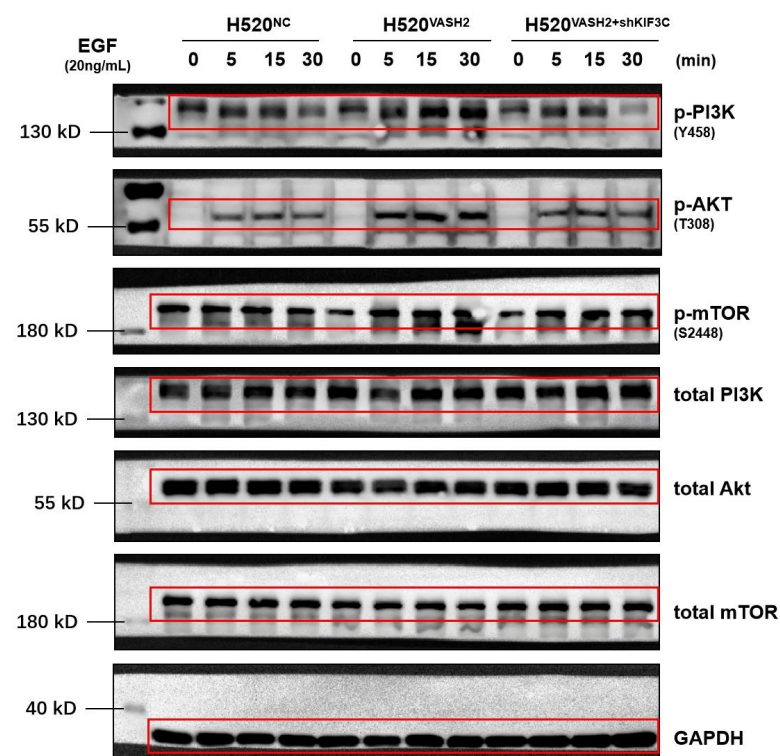

Fig. 6K

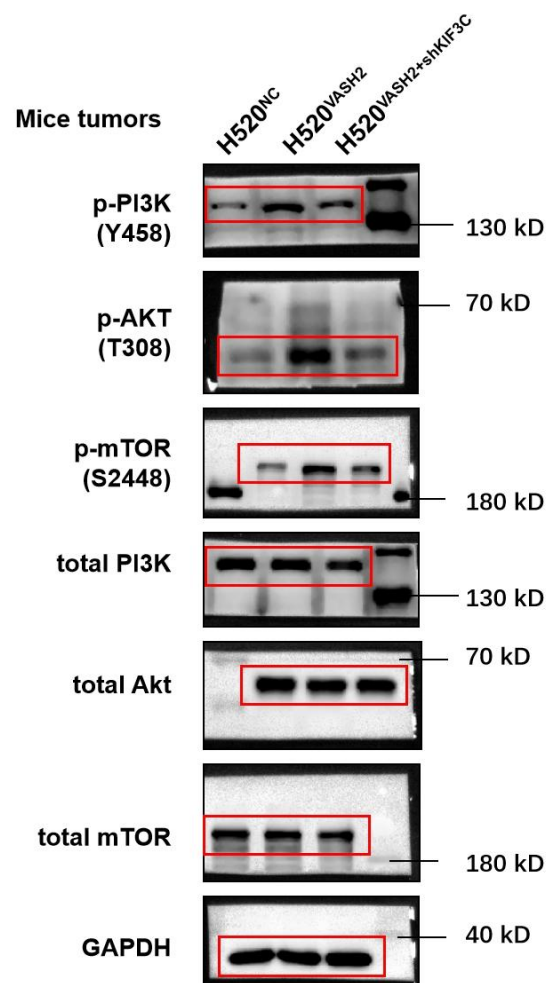

Supplement: Supplementary file 2 — Supplementary original WB data [file 41419_2024_7155_MOESM2_ESM.pdf]
